# Time-dependent efficacy analysis of first-line immunotherapies for advanced non–small cell lung cancer

**DOI:** 10.1186/s12885-024-12439-8

**Published:** 2024-06-05

**Authors:** Wen Hui, Wentan Li, Ruomeng Song, Yu Xin, Changjin Wu, Zhixiang Gao, Mingyue Zhang, Huazhang Wu, Min Zhu, Yuanyi Cai

**Affiliations:** 1 Department of Science and Techonology, West China Hospital, Sichuan University, Chengdu, 610041 China; 2 Department of Health Service Management, School of Health Management, China Medical University, Shenyang, 110122 China; 3 School of Public Health, China Medical University, Shenyang, 110122 China; 4 Department of Pharmacy, Affiliated Central Hospital of Shenyang Medical College, Shenyang, 110024 China; 5 Department of Health Economics, School of Health Management, China Medical University, Shenyang, 110122 China; 6 School of Medical Humanities, China Medical University, Shenyang, 110122 China

**Keywords:** Treatment effect, Non-small cell lung cancer, Immunotherapy, Hazard ratio, Programmed death-ligand 1

## Abstract

**Background:**

Many randomized controlled trials (RCTs) and network meta-analyses have demonstrated that the progression-free survival (PFS) and overall survival (OS) of advanced non-small cell lung cancer (NSCLC) patients can be improved through combination immunotherapy or monotherapies. However, time-dependent analysis of the treatment effect is currently lacking. Thus, we aimed to evaluate the efficacy of first-line immunotherapy, and establish a hazard ratio function to reflect the time-varying progression or mortality risk of patients with NSCLC.

**Methods:**

Seventeen clinical trials were selected based on search strategy. Baseline characteristics, including the age, sex, smoking status, geographical region, and Eastern Cooperative Oncology Group (ECOG) performance status of patients, were balanced, resulting in ten immunotherapies from nine appropriate clinical trials to conduct treatment effect comparison.

**Results:**

We found that nivolumab plus ipilimumab (nivo + ipi) improved the PFS and OS over time. The hazard ratio of nivo + ipi, relative to that of pembrolizumab, decreased from 1.11 to 0.36 for PFS, and from 0.93 to 0.49 for OS over a 10-year period. In terms of the response to immunotherapy in patients with different PD-L1 expression levels, patients with PD-L1 > = 50% experienced lower rates of progression and a reduced mortality risk over time. The hazard ratio of patients with PD-L1 > = 50% relative to all of the patients decreased from 0.73 to 0.69 for PFS, and from 0.78 to 0.67 for OS.

**Conclusions:**

Based on the fact that time-dependent progression and mortality risk existed during the treatment duration, physicians should select a suitable treatment regimen for patients based on the hazard ratio.

**Supplementary Information:**

The online version contains supplementary material available at 10.1186/s12885-024-12439-8.

## Background

Lung cancer is the leading cause of cancer-related deaths worldwide [1]. The 5-year survival rate of lung cancer is 21%, which is far lower than that of other cancers, such as melanoma (93%), breast cancer (90%), and prostate cancer (98%) [2]. Non-small cell lung cancer (NSCLC) is the main subtype of lung cancer, which constitutes approximately 85–90% of cases [3]. Coughing, which occurs in 50–75% of patients, is the most common symptom, followed by hemoptysis, chest pain, and dyspnea. Other symptoms include laboratory abnormalities and paraneoplastic syndromes [4]. Approximately 30–55% of patients who are diagnosed at an early stage and receive surgery experience recurrence [5]. Patients with NSCLC are often diagnosed at stages III and IV, with a low 5-year survival rate [6–8]. Thus, improving the survival rate of NSCLC is of the utmost relevance.

In the 20^th^ century, chemotherapy has become the fastest developing anti-tumor regimen in modern medicine. However, its efficacy and safety are well below patients’ expectations, which has led to the development of precision medicine, such as targeted therapy aimed at epidermal growth factor receptor (EGFR) and anaplastic lymphoma kinase (ALK). In recent years, the emergence of immunotherapies, such as pembrolizumab, nivolumab, atezolizumab, camrelizumab, sintilimab, and tislelizumab, has greatly extended the survival of advanced patients without sensitizing them to EGFR or ALK mutations. In addition to monotherapy, combination immunotherapy regimens have also been extensively explored.

The detailed effects of immunotherapy treatment has already been presented in a series of randomized controlled trials (RCT), such as KEYNOTE-189, CheckMate-9LA, IMpower-130, CameL, ORIENT-11, and RATIONALE-304, demonstrating the improvement of patients’ progression-free survival (PFS) and overall survival (OS), compared with that observed after chemotherapy [9–14]. More precise meta-analyses have also demonstrated the improved clinical efficacy of immunotherapies. For example, a published network meta-analysis involving 10 immunotherapy combinations showed that pembrolizumab plus chemotherapy was comparable with sintilimab plus chemotherapy in terms of OS (hazard ratio (HR) = 0.96), and atezolizumab plus bevacizumab plus chemotherapy was found to provide the best PFS benefit compared with chemotherapy (HR = 0.45) [15]. However, the time-varying treatment effects of immunotherapies in patients with different PD-L1 expression levels in these RCTs or network meta-analyses were not thoroughly considered, indicating that the hazard ratio was stationary.

In our study, we aimed to analyze the efficacy of immunotherapy for NSCLC, and establish the hazard ratio function for immunotherapy to reflect time-dependent progression or mortality risk to aid physicians and researchers in obtaining a comprehensive assessment of the effects of immunotherapy over time.

## Materials and methods

### Materials

All the data used in our study were from published RCTs. Through reconstruction of individual patient data in these clinical trials, datasets were formed to compare the treatment effect of immunotherapies.

### Search strategy and selection procedure

Data retrieval was performed between 2021 and 2023 using Pubmed and Web of Science. The following keywords were used: “lung cancer” and “network meta-analysis”. Sixteen network meta-analyses, including 39 clinical trials, were included in further selection procedures. Two rounds of screening were conducted for the 39 clinical trials. The first round of screening included titles and abstracts and was based on the exclusion criteria, including subsequent-line therapy, phase II clinical trials, and squamous NSCLC for histology. In the second step, the selected articles were further evaluated through full-text reading, and based on the following exclusion criteria: not targeting programmed death one (PD-1) or programmed death ligand one (PD-L1), and no significant efficacy compared with chemotherapy. Seventeen clinical trials were included after the selection (Figure S1).

### Baseline characteristics comparison

Baseline characteristics, including the age, sex, smoking status, geographical region, and Eastern Cooperative Oncology Group (ECOG) performance status for patients randomized into recruited experimental groups in 17 clinical trials, were compared [9–14, 16–26]. To avoid bias due to unbalanced baseline characteristics, the following criteria were defined (Figure S2, Table S1-S2).

1. Age: The number of patients with an age greater than or equal to 65 years old constituted 50% ± 10% of patients. Clinical trials, including CameL, RATIONALE-304, and GEMSTONE-302, were excluded.
2. Sex: Male patients constituted 70% ± 10%. EMPOWER-Lung 1 was excluded.
3. Smoking status: Non-smokers constituted 15% ± 10% of patients. KEYNOTE-024, ORIENT-11, and CHOICE-01 were excluded.
4. Race or Geographical region: The patients in the experimental groups were mainly Caucasian or from North America and Europe (after the three above steps), except for TASUKI-52, which was excluded.
5. ECOG performance status: The number of patients with an ECOG ≧1 constituted 60% ± 10%. None of the clinical trials were excluded in this step. Finally, nine clinical trials, including CheckMate-9LA, CheckMate-227 Part 1, KEYNOTE-189, KEYNOTE-042, KEYNOTE-598, IMpower-110, IMpower-130, IMpower-132, and IMpower-150, were used to conduct efficacy analysis. In the nine clinical trials, there were ten immunotherapy regimens and four PD-L1 expression levels. The immunotherapy regimens included nivolumab plus ipilimumab plus chemotherapy (nivo + ipi + chemo), nivolumab (nivo), nivolumab plus ipilimumab (nivo + ipi), nivolumab plus chemotherapy (nivo + chemo), atezolizumab (ate), atezolizumab plus chemotherapy (ate + chemo), atezolizumab plus bevacizumab plus chemotherapy (ate + beva + chemo), pembrolizumab plus chemotherapy (pem + chemo), pembrolizumab plus ipilimumab (pem + ipi), and pembrolizumab (pem). The PD-L1 expression levels were as follows: PD-L1 ≥ 1%, PD-L1 ≥ 1% and < 50%, PD-L1 ≥ 50%, and PD-L1 < 1%.

### Reconstruction of individual patient data

WebPlotDigitizer was used to obtain individual patient data (IPD) of PFS and OS curves in ten immunotherapy arms with four PD-L1 expression levels from nine clinical trials [10, 11, 19–22, 27–30]. Long-term follow-up endpoint in a clinical trial was preferentially considered, if the data was published. Primary results were also included for obtaining specific survival information. The reconstructed IPD were further organized by adding the number of events and the number of patients at risk (Table S3 and S4).

### Statistical analysis

R software (V4.2.2) was used for data analysis. The time-varying hazard functions of 10 immunotherapy regimens with four PD-L1 expression levels were established, based on the following derivation [31–33]:

$$\begin{aligned}P_{jkt}\;&=\frac{S(t-\Delta t)-S(t)}{S(t-\Delta t)}\\&=1-e^-\int_{t-\Delta t}^th(u)du\cdot\cong1-\text e^{-\Delta \text t\ast \text h_ \text{jkt}}\cdot(\Delta t\rightarrow0),\end{aligned}$$

where *p*_*jkt*_ is the event probability for PD-L1 level k in treatment j at time t. h_jkt_ can be transformed as follows,

$$h_{jkt}\cong-\ln\left(1-p_{jkt}\right)/\triangle t_{jkt}$$, namely,

ln $\left(h_{jkt}\right)\cong c\log\log\left(p_{jkt}\right)-\ln\left(\triangle t_{jkt}\right)\;=\;\eta_{jkt.}\;\eta_{jkt}$ is the linear predictor, and is defined as,

$$\eta jkt={\textstyle\sum_{m=0}^M}\left(\alpha_{mj}+\theta_{mk}\right)\ast gm(t),$$

where $\alpha_{mj}$ are the treatment-specific coefficients for treatment strategy j, the $\theta_{m\text k}$ are the PD-L1 expression-specific coefficients for PD-L1 expression level k, and gm(t) are a set of functions. In this study, it was set to gm(t) = t^pm^, which was consistent with the fractional polynomial (FP) model. The first-order FP model is defined as,

$$\gamma=\beta_{\mathit0}+\beta_{\mathit1}t^p$$

The power p is selected from the following set: -2, -1,-0.5, 0, 0.5, 1, 2, 3 with *t*^0^ = log *t.* The second order FP is defined as,

$$\gamma=\beta_{\mathit0}+\beta_{\mathit1}t^{\mathit p\mathit1}+\beta_{\mathit2}t^{p\mathit2}$$

If p1 = p2 = p, the model is defined as,

$$\gamma\:=\:\beta_{\mathit0}\:+\:\beta_{\mathit1}t^{\mathit p}\mathit\:+\:\beta_2t^p\mathrm{logt}$$

Therefore, according to the power p, one model from the eight first-order FP models and thirty-six second order FP models needed to be found to best fit η_jkt._ According to the Akaike information criterion (AIC), and visual inspection, the FP model with power p1 = -2, and p2 = -1 was the best fit for PFS, and the FP model with power p1 = -2, and p2 = 0 was the best fit for OS (Table S5-S6, Fig.S3-S6). When the treatment effects of ten immunotherapy regimens were compared, “pem” was the reference, and “all the patients” was the value of controlled variable. When four PD-L1 effects were compared, “all the patients” was the reference, and “pem” was the value of controlled variable.

## Results

### Progression-free survival

Based on the second order FP with power p1 = -2, and p2 = -1, the hazard function of PFS for each of the ten immunotherapy regimens and each of the four PD-L1 expression levels was presented in Table S7-S8.

**Fig. 1:**
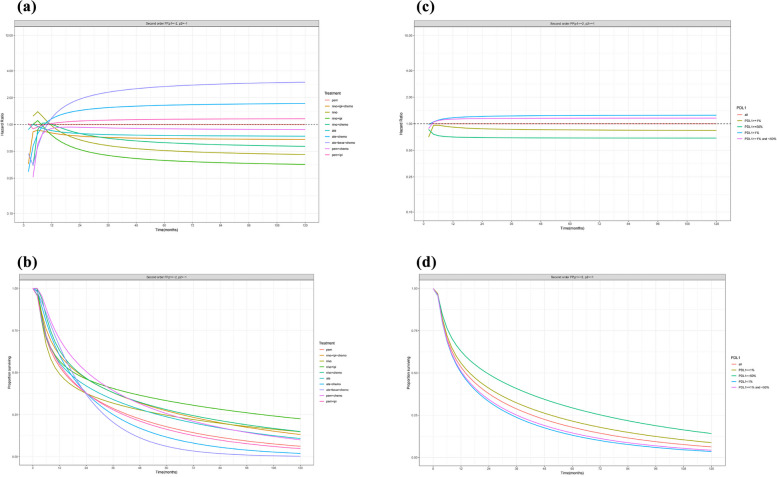
Treatment effect for PFS over time (**A**) Hazard ratio of PFS over time for each of the immunotherapies relative to pem as obtained with second order fractional polynomial (p1 = -2, p2 = -1) (**B**) PFS over time for each of the immunotherapies as obtained with second order fractional polynomial (p1 = -2, p2 = -1) (**C**) Hazard ratio of PFS over time for each of the 4 PD-L1 expression levels relative to all the patients as obtained with second order fractional polynomial (p1 = -2, p2 = -1) (**D**) PFS over time for each of the 4 PD-L1 expression levels as obtained with second order fractional polynomial (p1 = -2, p2 = -1)

Regarding the treatment effect, the corresponding time-varying hazard ratio of each immunotherapy regimen relative to pem is shown in Table 1. We found that nivo monotherapy and combination therapy provided better PFS benefit over time. For example, although the hazard ratio of nivo + ipi relative to pem was 1.11 after 6 months, it steadily declined in the following years, and decreased to 0.36 after 10 years (Fig. 1).

**Table 1:** Hazard ratios of PFS for 9 treatment strategies relative to pem

	Treatment	HR(t)	6 month	1 year	2 year	3 year	5 year	10 year
1	nivo + ipi + chemo vs. pem	e ^-0.4116 - 8.4512t-2+3.0029t-1^	0.86	0.8	0.74	0.72	0.69	0.68
2	nivo vs. pem	e ^-0.8817 - 34.6902t-2+13.06t-1^	1.39	0.97	0.67	0.58	0.51	0.46
3	nivo + ipi vs. pem	e ^-1.1388 - 34.3352t-2+13.1567t-1^	1.11	0.76	0.52	0.45	0.39	0.36
4	nivo + chemo vs. pem	e ^-0.6673 - 57.7548t-2+12.84t-1^	0.88	1	0.79	0.7	0.63	0.57
5	ate vs. pem	e ^-0.3209 - 3.7536t-2+2.2513t-1^	0.95	0.85	0.79	0.77	0.75	0.74
6	ate + chemo vs. pem	e ^0.5887 + 3.9319t-2-5.5853t-1^	0.79	1.16	1.44	1.55	1.64	1.72
7	ate + beva + chemo vs. pem	e ^1.21 + 20.4349t-2-14.1401t-1^	0.56	1.19	1.93	2.3	2.66	2.99
8	pem + chemo vs. pem	e ^-0.1593 - 33.1034t-2+3.4654t-1^	0.61	0.9	0.93	0.92	0.9	0.88
9	pem + ipi vs. pem	e ^0.1648 + 3.3639t-2-1.9485t-1^	0.94	1.03	1.09	1.12	1.14	1.16

Regarding the PD-L1 effect, Table 2 presents the corresponding time-varying hazard ratio of patients with each PD-L1 expression level relative to all of the patients. Patients with PD-L1 ≥ 50% experienced lower progression risk over time. The hazard ratios of patients with PD-L1 ≥ 50% relative to all patients were maintained at lower levels: 0.73 at 6 months to 0.69 at 10 years (Fig. 1).

**Table 2:** Hazard ratios of PFS for patients with 4 PD-L1 expression levels relative to all the patients

	PD-L1	HR(t)	6 month	1 year	2 year	3 year	5 year	10 year
1	> = 1% vs. all	${\mathrm e}^{-0.183 - 3.5433{\mathrm t}^{-2} + 1.4457{\mathrm t}^{-1}}$	0.96	0.92	0.88	0.86	0.85	0.84
2	> = 1% and < 50% vs. all	e ^0.1493 - 0.4858t-2-0.3008t-1^	1.09	1.13	1.15	1.15	1.16	1.16
3	> = 50% vs. all	e ^-0.3763 + 0.1784t-2+0.365t-1^	0.73	0.71	0.7	0.69	0.69	0.69
4	< 1% vs. all	e ^0.2315 + 0.9136t-2-0.9591t-1^	1.1	1.17	1.21	1.23	1.24	1.25

### Overall survival

Based on the second order FP with power p1 = -2, and p2 = 0, the hazard function of OS for each of the ten immunotherapy regimens and each of the four PD-L1 expression levels is shown in Table S9-S10.

**Fig. 2:**
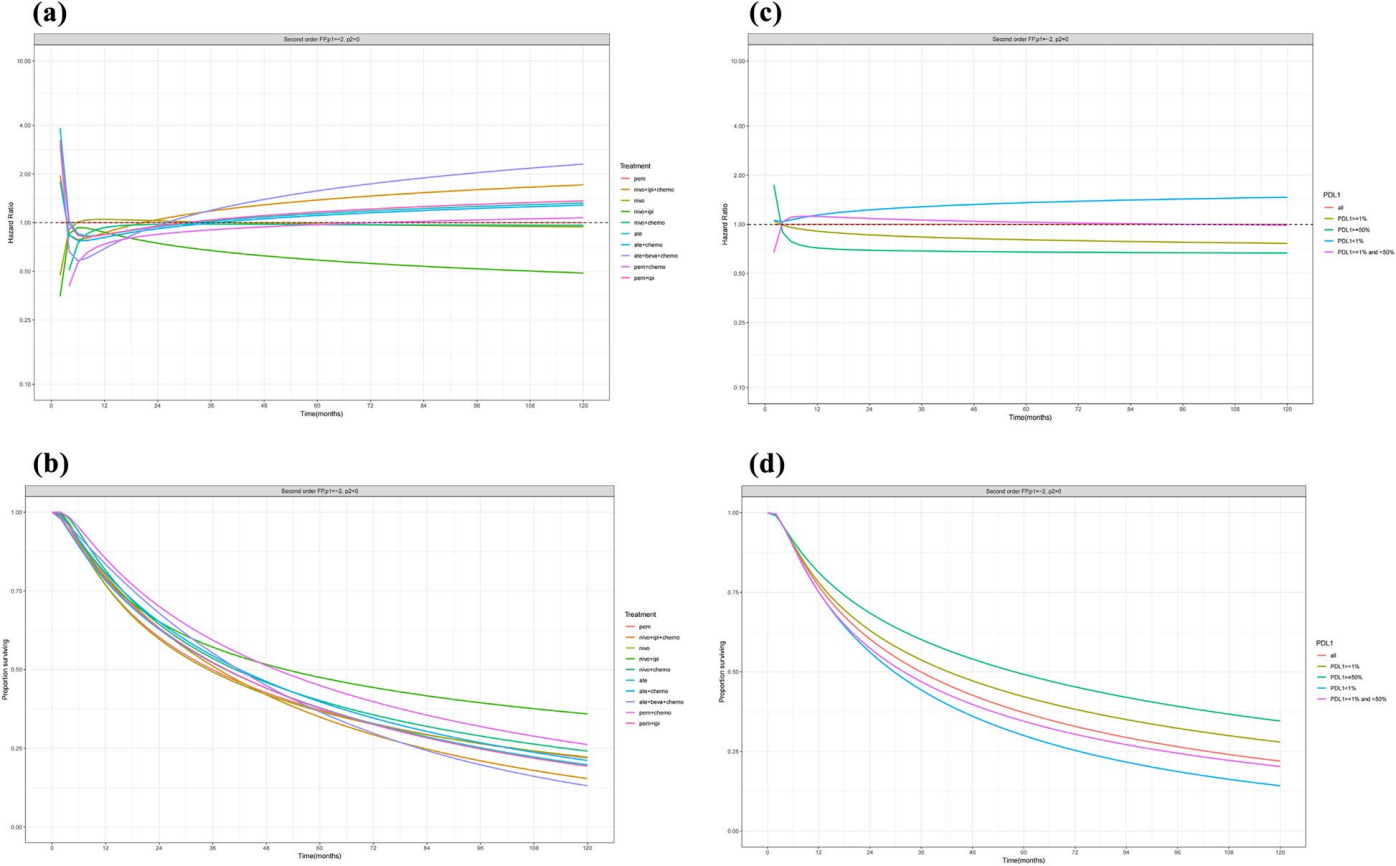
Treatment effect for OS over time (**A**) Hazard ratio of OS over time for each of the immunotherapies relative to pem as obtained with second order fractional polynomial (p1 = -2, p2 = 0) (**B**) OS over time for each of the immunotherapies as obtained with second order fractional polynomial (p1 = -2, p2 = 0) (**C**) Hazard ratio of OS over time for each of the 4 PD-L1 expression levels relative to all the patients as obtained with second order fractional polynomial (p1 = -2, p2 = 0) (**D**) OS over time for each of the 4 PD-L1 expression levels as obtained with second order fractional polynomial (p1 = -2, p2 = 0)

In terms of the treatment effect, among the ten treatment strategies, only nivo + ipi resulted in a better overall survival, with hazard ratios of nivo + ipi relative to pem at 6 months, 1 year, 2 years, 3 years, 5 years, and 10 years of 0.93, 0.87, 0.75, 0.67, 0.59, and 0.49, respectively. The other eight treatment strategies presented higher or similar mortality risks compared with that of pem (Table 3, Fig. 2).

**Table 3:** Hazard ratios of OS for 9 treatment strategies relative to pem

	Treatment	HR(t)	6 month	1 year	2 year	3 year	5 year	10 year
1	nivo + ipi + chemo vs. pem	${\mathrm e}^{-0.96118 + 5.66895{\mathrm t}^{-2} + 0.31327 \text{log}{\mathrm (\text t)}}$	0.78	0.87	1.05	1.18	1.38	1.71
2	nivo vs. pem	e ^0.21729 - 3.709t-2-0.0578log(t)^	1.01	1.05	1.03	1.01	0.98	0.94
3	nivo + ipi vs. pem	e ^0.57486 - 5.7528t-2-0.26978log(t)^	0.93	0.87	0.75	0.67	0.59	0.49
4	nivo + chemo vs. pem	e ^0.05961 - 11.37581t-2-0.0202log(t)^	0.75	0.93	0.98	0.98	0.97	0.96
5	ate vs. pem	e ^-0.76807 + 7.85156t-2+0.218649log(t)^	0.85	0.84	0.94	1.02	1.14	1.32
6	ate + chemo vs. pem	e ^-0.77457 + 4.85285t-2+0.21399log(t)^	0.77	0.81	0.92	1	1.11	1.28
7	ate + beva + chemo vs. pem	e ^-1.81551 + 10.21466t-2+0.5535log(t)^	0.58	0.69	0.96	1.19	1.57	2.3
8	pem + chemo vs. pem	e ^-0.59121 - 8.09756t-2+0.13829log(t)^	0.57	0.74	0.85	0.9	0.97	1.07
9	pem + ipi vs. pem	e ^-0.79555 + 7.24569t-2+0.23109log(t)^	0.84	0.84	0.95	1.04	1.16	1.37

In terms of the PD-L1 effect, a significantly improved OS was observed in the higher PD-L1 expression level. The hazard ratio of patients with PD-L1 ≥ 50% relative to all patients decreased from 0.78 to 0.67 over time, followed by PD-L1 ≥ 1% (hazard ratio interval: 0.76–0.96). Patients with PD-L1 < 1% were associated with higher mortality risk, which increased over time, from 1.07 at 6 months to 1.46 at 10 years (Table 4, Fig. 2).

**Table 4:** Hazard ratios of OS for patients with 4 PD-L1 expression levels relative to all the patients

	PD-L1	HR(t)	6 month	1 year	2 year	3 year	5 year	10 year
1	> = 1% vs. all	e ^0.08777 + 0.05423t-2-0.07439log(t)^	0.96	0.91	0.86	0.84	0.81	0.76
2	> = 1% and < 50% vs. all	e ^0.28072 - 2.54688t-2-0.06103log(t)^	1.11	1.12	1.09	1.06	1.03	0.99
3	> = 50% vs. all	e ^-0.3031 + 3.506t-2-0.02146log(t)^	0.78	0.72	0.69	0.69	0.68	0.67
4	< 1% vs. all	e ^-0.14963 + 0.51444t-2+0.111log(t)^	1.07	1.14	1.23	1.28	1.36	1.46

## Discussion

In our research, we conducted a quantitative analysis and reported the hazard ratio function to reflect the time-varying treatment effects of various immunotherapy regimens and subtypes of PD-L1 expression levels. The baseline characteristics, including the age, sex, smoking status, geographical region, and ECOG performance status, of patients using immunotherapies in the nine clinical trials, were balanced. PFS and OS curves for patients with different PD-L1 expression levels were extracted, indicating that information from a single RCT was used to the maximum extent to support a more precise progression or mortality risk analysis. Based on the IPD from RCTs, the best model was selected from 44 FPs to conduct regression analysis. The hazard ratio functions displayed the existence of a time-varying treatment effect. This finding serves as a basis for physicians and researchers to further explore the treatment effects of immunotherapies over time, and comprehensively understand the dynamic changes between the tumor and the patient's immune system.

Previous studies have shown that nivo + ipi or patients with PD-L1 ≥ 50% exhibit a better survival benefit [15, 27, 34], based on the constant hazard ratio. Our study revealed similar findings, and further evaluated how this advantage of nivo + ipi treatment or patients with PD-L1 ≥ 50% varied over time. We found that this advantage gradually started after 12 months (PFS) or 18 months (OS) for nivo + ipi, and just after 3 months (OS and PFS) for patients with PD-L1 ≥ 50%.

The differences in the time-varying efficacy of different immunotherapies are mainly due to the patient’s biological characteristics, which play an important role in the selection of immunotherapy. Whether immunotherapy is chosen (monotherapy or immunotherapy combination) or not, the efficacy and duration of the effect highly depend on the biomarkers existing in patients. Although a substantial amount of work has been conducted, more researches are required to uncover the mechanisms underlying the response to immune checkpoint inhibitors in patients with NSCLC, which will be beneficial for personalized immunotherapy [35, 36].

Although the treatment effect was the focus of our study, the safety and economic burden of immunotherapy should not be neglected. Factors such as patients’ age, ECOG status, and tolerance to platinum-based chemotherapy may affect the use of immunotherapy regimens. If grade ≥ 3 adverse events frequently occur, physicians may have to discontinue immunotherapy or alter treatment regimens [37]. However, its safety remains controversial. For example, immune-related adverse events may be associated with better antineoplastic activity [38], which requires physicians to balance efficacy and safety for patients. Economic affordability should also be considered. Previous health technology assessment of immunotherapy combinations revealed that nivolumab plus ipilimumab was cost-effective in the United States, but the economic advantage was not achieved in China [39, 40].

Time-varying progression or mortality risk analyses will provide further support for future research. First, it is invalid in some patients after immunotherapy. This rapid progression or mortality should be traced. Second, drug resistance also occurs during immunotherapy, which requires investigations into the mechanism and control of the time point of occurrence. Third, the period at which immunotherapy needs to last, and whether or not the patients stop immunotherapy after two years, is an intractable problem for physicians.

There are a few limitations in our study. First, the PD-L1 expression level was divided into ≥ 1%, ≥ 1% and < 50%, ≥ 50%, and < 1% in our study, while other PD-L1 expression levels, such as 50–89% and > 90% were not considered, due to the lack of corresponding information in the clinical trials. A recent study on patients using pembrolizumab indicated that the median PFS was 9.0 months in the PD-L1 > 90% group, and 5.4 months in the 50–89% group, and the median OS was 30.4 months vs. 18.6 months, respectively [41]. There is a lack of direct comparisons among immunotherapies in the nine clinical trials used in our study. Notably, the direct comparison has started in current clinical trials. For example, a randomized, phase 2 trial (CTONG1901) directly compared the efficacy of sintilimab and pembrolizumab as a treatment for patients with NSCLC [42]. If direct evidence is used, the uncertainty of the comparison decreases. Third, the result of PD-L1 expression in each clinical trial was directly used in our research. However, it could be affected by using different PD-L1 assay [43]. Fourth, although baseline characteristics, including the age, sex, smoking status, geographical region, and ECOG performance status were balanced, other characteristics, such as brain or liver metastases at baseline, were not adjusted due to the absence of information.

## Conclusions

In this research, we evaluated the time-varying treatment effects of ten immunotherapies in patients with four PD-L1 expression levels. Our findings showed that time-dependent progression and mortality risk exist during the treatment. This serves as evidence that may aid physicians in choosing a suitable immunotherapy regimen for patients based on its short- and long-term treatment effects.

### Supplementary Information

Supplementary Material 1.

## Data Availability

Data is provided within the manuscript or supplementary information files.

## Abbreviations

NSCLC: Non-small cell lung cancer
EGFR: Epidermal growth factor receptor
ALK: Anaplastic lymphoma kinase
RCT: Randomized controlled trial
PFS: Progression-free survival
OS: Overall survival
HR: Hazard ratio
PD-1: Programmed death one
PD-L1: Programmed death ligand one
ECOG: Eastern cooperative oncology group
nivo + ipi + chemo: Nivolumab plus ipilimumab plus chemotherapy
nivo: Nivolumab
nivo + ipi: Nivolumab plus ipilimumab
nivo + chemo: Nivolumab plus chemotherapy
ate: Atezolizumab
ate + chemo: Atezolizumab plus chemotherapy
ate + beva + chemo: Atezolizumab plus bevacizumab plus chemotherapy
pem + chemo: Pembrolizumab plus chemotherapy
pem + ipi: Pembrolizumab plus ipilimumab
pem: Pembrolizumab
IPD: Individual patient data
AIC: Akaike information criterion
