# Supplementary material for: Time-dependent efficacy analysis of first-line immunotherapies for advanced non–small cell lung cancer
Source: BMC Cancer. 2024 Jun 5;24:684. doi: 10.1186/s12885-024-12439-8 (PMC11151632; doi:10.1186/s12885-024-12439-8)
Supplement: Supplementary file 1 — Supplementary Material 1. [file 12885_2024_12439_MOESM1_ESM.pdf]

Time-dependent efficacy analysis of first-line immunotherapies for advanced non − small cell lung cancer

Wen Hui, Wentan Li, Ruomeng Song, Yu Xin, Changjin Wu, Zhixiang Gao, Mingyue Zhang, Huazhang Wu, Min Zhu, Yuanyi Cai

Supplementary Materials

Title	Content	Page
Table S1	Unbalanced Baseline Characteristics in the 17 Clinical Trials	2-3
Table S2	Balanced Baseline Characteristics in the 9 Clinical Trials	4
Table S3	Dateset of PFS Used to Fractional Polynomial Analysis	5-22
Table S4	Dateset of OS Used to Fractional Polynomial Analysis	23-47
Table S5	AIC Value for PFS Fit	48-49
Table S6	AIC Value for OS Fit	50-51
Table S7	Hazard Function of PFS for Each of the 10 Immunotherapy Regimens	52
Table S8	Hazard Function of PFS for Each of the 4 PD-L1 Expression Levels	53
Table S9	Hazard Function of OS for Each of the 10 Immunotherapy Regimens	54
Table S10	Hazard Function of OS for Each of the 4 PD-L1 Expression Levels	55
Figure S1	Flow diagram for the clinical trial selection procedure	56
Figure S2	Flow diagram for the baseline characteristic balance	57
Figure S3	Visual Inspection of 6 PFS Second Order Fractional Polynomial Models among 10 Immunotherapies	58
Figure S4	Visual Inspection of 6 PFS Second Order Fractional Polynomial Models among 4 PD-L1 expression levels	59
Figure S5	Visual Inspection of 8 OS Second Order Fractional Polynomial Models among 10 Immunotherapies	60
Figure S6	Visual Inspection of 8 OS Second Order Fractional Polynomial Models among 4 PD-L1 expression levels	61

Table S1 Unbalanced baseline characteristics in the 17 clinical trials

Study	Intervention Arm(s)	Control Arm(s)	Age	Sex	Smoking	Race/ region	ECOG
CheckMate-9LA	Nivolumab + Ipilimumab + Chemotherapy	Chemotherapy	0.51	0.7	0.13	0.69	0.68
CheckMate-227 part 1	Nivolumab + Ipilimumab	Chemotherapy	0.475	0.674	0.136	0.792	0.65
	Nivolumab	Chemotherapy	0.47	0.687	0.126	0.833	0.636
	Nivolumab+ Chemotherapy	Chemotherapy	0.486	0.734	0.153	0.796	0.661
KEYNOTE-189	Pembrolizumab + Chemotherapy	Chemotherapy	0.52	0.62	0.117	0.864	0.541
KEYNOTE-024	Pembrolizumab	Chemotherapy	-	0.597	0.032	0.864	0.649
KEYNOTE-042	Pembrolizumab	Chemotherapy	0.44	0.71	0.22	0.71	0.69
KEYNOTE-598	Pembrolizumab + Ipilimumab	-	0.482	0.711	0.102	0.887	0.644
	-	Pembrolizumab+ Placebo	0.528	0.673	0.088	0.891	0.634
Impower-110	Atezolizumab	Chemotherapy	0.484	0.708	0.134	0.819	0.65
Impower-130	Atezolizumab + Chemotherapy	Chemotherapy	0.5	0.59	0.11	0.89	0.58
Impower-132	Atezolizumab + Chemotherapy	Chemotherapy	0.476	0.658	0.127	0.661	0.568
Impower-150	Atezolizumab + Bevacizumab + Chemotherapy	Bevacizumab + Chemotherapy	0.462	0.6	0.205	0.805	0.599
	Atezolizumab + Chemotherapy	Bevacizumab + Chemotherapy	0.445	0.6	0.192	0.823	0.552
CameL	Camrelizumab + Chemotherapy	Chemotherapy	0.22	0.71	0.36	0	0.77
RATIONALE-304	Tislelizumab + Chemotherapy	Chemotherapy	0.269	0.753	0.341	0	0.758
ORIENT-11	Sintilimab + Chemotherapy	Chemotherapy	0.526*	0.767	0.357	0	0.714
EMPOWER-Lung 1	Cemiplimab	Chemotherapy	0.45	0.88	0	0.76	0.73

Gemstone-302	Sugemalimab+ Chemotherapy	Chemotherapy	0.37	0.79	0.27	0	0.82
CHOICE-01	Toripalimab + Chemotherapy	Chemotherapy	0.421	0.799	0.311	0	0.786
TASUKI-52	Nivolumab + Bevacizumab + Chemotherapy	Bevacizumab + Chemotherapy	0.524	0.745	0.222	0	0.531

* In ORIENT-11 trial, it was the percentage of greater than 60 years old.

Table S2 Balanced baseline characteristics in the 9 clinical trials

Study	Intervention Arm(s)	Control Arm(s)	Age	Sex	Smoking	Race/ region	ECOG
CheckMate-9LA	Nivolumab + Ipilimumab + Chemotherapy	Chemotherapy	0.51	0.7	0.13	0.69	0.68
CheckMate-227 part 1	Nivolumab + Ipilimumab	Chemotherapy	0.475	0.674	0.136	0.792	0.65
	Nivolumab	Chemotherapy	0.47	0.687	0.126	0.833	0.636
	Nivolumab+ Chemotherapy	Chemotherapy	0.486	0.734	0.153	0.796	0.661
KEYNOTE-189	Pembrolizumab + Chemotherapy	Chemotherapy	0.52	0.62	0.117	0.864	0.541
KEYNOTE-042	Pembrolizumab	Chemotherapy	0.44	0.71	0.22	0.71	0.69
KEYNOTE-598	Pembrolizumab + Ipilimumab	-	0.482	0.711	0.102	0.887	0.644
	-	Pembrolizumab+ Placebo	0.528	0.673	0.088	0.891	0.634
Impower-110	Atezolizumab	Chemotherapy	0.484	0.708	0.134	0.819	0.65
Impower-130	Atezolizumab + Chemotherapy	Chemotherapy	0.5	0.59	0.11	0.89	0.58
Impower-132	Atezolizumab + Chemotherapy	Chemotherapy	0.476	0.658	0.127	0.661	0.568
Impower-150	Atezolizumab + Bevacizumab + Chemotherapy	Bevacizumab + Chemotherapy	0.462	0.6	0.205	0.805	0.599
	Atezolizumab + Chemotherapy	Bevacizumab + Chemotherapy	0.445	0.6	0.192	0.823	0.552

Table S3 Dateset of PFS used to fractional polynomial analysis

Study	Treatment	PD-L1	Time	Time Interval	No.Events	No.at Risk
CM9LA	NIVO+IPI+chemo	all	2	2	13	361
CM9LA	NIVO+IPI+chemo	all	4	2	73	307
CM9LA	NIVO+IPI+chemo	all	6	2	43	210
CM9LA	NIVO+IPI+chemo	all	8	2	25	161
CM9LA	NIVO+IPI+chemo	all	10	2	19	136
CM9LA	NIVO+IPI+chemo	all	12	2	15	117
CM9LA	NIVO+IPI+chemo	all	14	2	10	102
CM9LA	NIVO+IPI+chemo	all	16	2	14	92
CM9LA	NIVO+IPI+chemo	all	18	2	5	78
CM9LA	NIVO+IPI+chemo	all	20	2	5	73
CM9LA	NIVO+IPI+chemo	all	22	2	5	68
CM9LA	NIVO+IPI+chemo	all	24	2	4	62
CM9LA	NIVO+IPI+chemo	all	26	2	5	54
CM9LA	NIVO+IPI+chemo	all	28	2	1	36
CM9LA	NIVO+IPI+chemo	all	30	2	3	24
CM9LA	NIVO+IPI+chemo	all	32	2	0	12
CM9LA	NIVO+IPI+chemo	all	34	2	0	8
CM9LA	NIVO+IPI+chemo	all	36	2	0	4
CM9LA	NIVO+IPI+chemo	all	38	2	0	1
CM9LA	NIVO+IPI+chemo	>=1%	2	2	4	204
CM9LA	NIVO+IPI+chemo	>=1%	4	2	42	176
CM9LA	NIVO+IPI+chemo	>=1%	6	2	24	120
CM9LA	NIVO+IPI+chemo	>=1%	8	2	15	94
CM9LA	NIVO+IPI+chemo	>=1%	10	2	11	79
CM9LA	NIVO+IPI+chemo	>=1%	12	2	10	68
CM9LA	NIVO+IPI+chemo	>=1%	14	2	4	58
CM9LA	NIVO+IPI+chemo	>=1%	16	2	11	54
CM9LA	NIVO+IPI+chemo	>=1%	18	2	3	43
CM9LA	NIVO+IPI+chemo	>=1%	20	2	1	40
CM9LA	NIVO+IPI+chemo	>=1%	22	2	2	39
CM9LA	NIVO+IPI+chemo	>=1%	24	2	4	37
CM9LA	NIVO+IPI+chemo	>=1%	26	2	1	32
CM9LA	NIVO+IPI+chemo	>=1%	28	2	1	22
CM9LA	NIVO+IPI+chemo	>=1%	30	2	1	14
CM9LA	NIVO+IPI+chemo	>=1%	32	2	0	7
CM9LA	NIVO+IPI+chemo	>=1%	34	2	0	4
CM9LA	NIVO+IPI+chemo	>=1%	36	2	0	2
CM9LA	NIVO+IPI+chemo	>=1%	38	2	0	0
CM9LA	NIVO+IPI+chemo	>=50%	2	2	0	76
CM9LA	NIVO+IPI+chemo	>=50%	4	2	17	65
CM9LA	NIVO+IPI+chemo	>=50%	6	2	8	43

CM9LA	NIVO+IPI+chemo	>=50%	8	2	3	33
CM9LA	NIVO+IPI+chemo	>=50%	10	2	3	30
CM9LA	NIVO+IPI+chemo	>=50%	12	2	3	27
CM9LA	NIVO+IPI+chemo	>=50%	14	2	2	24
CM9LA	NIVO+IPI+chemo	>=50%	16	2	3	22
CM9LA	NIVO+IPI+chemo	>=50%	18	2	1	19
CM9LA	NIVO+IPI+chemo	>=50%	20	2	1	18
CM9LA	NIVO+IPI+chemo	>=50%	22	2	0	17
CM9LA	NIVO+IPI+chemo	>=50%	24	2	1	17
CM9LA	NIVO+IPI+chemo	>=50%	26	2	0	16
CM9LA	NIVO+IPI+chemo	>=50%	28	2	0	12
CM9LA	NIVO+IPI+chemo	>=50%	30	2	1	8
CM9LA	NIVO+IPI+chemo	>=50%	32	2	0	4
CM9LA	NIVO+IPI+chemo	>=50%	34	2	0	2
CM9LA	NIVO+IPI+chemo	>=50%	36	2	0	1
CM9LA	NIVO+IPI+chemo	>=50%	38	2	0	0
CM9LA	NIVO+IPI+chemo	<1%	2	2	5	135
CM9LA	NIVO+IPI+chemo	<1%	4	2	29	112
CM9LA	NIVO+IPI+chemo	<1%	6	2	14	74
CM9LA	NIVO+IPI+chemo	<1%	8	2	12	58
CM9LA	NIVO+IPI+chemo	<1%	10	2	6	45
CM9LA	NIVO+IPI+chemo	<1%	12	2	6	39
CM9LA	NIVO+IPI+chemo	<1%	14	2	4	33
CM9LA	NIVO+IPI+chemo	<1%	16	2	2	29
CM9LA	NIVO+IPI+chemo	<1%	18	2	0	27
CM9LA	NIVO+IPI+chemo	<1%	20	2	2	27
CM9LA	NIVO+IPI+chemo	<1%	22	2	3	25
CM9LA	NIVO+IPI+chemo	<1%	24	2	1	22
CM9LA	NIVO+IPI+chemo	<1%	26	2	1	19
CM9LA	NIVO+IPI+chemo	<1%	28	2	0	13
CM9LA	NIVO+IPI+chemo	<1%	30	2	2	10
CM9LA	NIVO+IPI+chemo	<1%	32	2	0	5
CM9LA	NIVO+IPI+chemo	<1%	34	2	0	4
CM9LA	NIVO+IPI+chemo	<1%	36	2	0	3
CM9LA	NIVO+IPI+chemo	<1%	38	2	0	1
CM227	NIVO	>=1% and <50%	2	2	0	182
CM227	NIVO	>=1% and <50%	4	2	69	166
CM227	NIVO	>=1% and <50%	6	2	22	82
CM227	NIVO	>=1% and <50%	8	2	14	42
CM227	NIVO	>=1% and <50%	10	2	5	28
CM227	NIVO	>=1% and <50%	12	2	5	23
CM227	NIVO	>=1% and <50%	14	2	4	18
CM227	NIVO	>=1% and <50%	16	2	3	14
CM227	NIVO	>=1% and <50%	18	2	1	11

CM227	NIVO	>=1% and <50%	20	2	1	10
CM227	NIVO	>=1% and <50%	22	2	2	9
CM227	NIVO	>=1% and <50%	24	2	0	7
CM227	NIVO	>=1% and <50%	26	2	0	7
CM227	NIVO	>=1% and <50%	28	2	0	7
CM227	NIVO	>=1% and <50%	30	2	1	6
CM227	NIVO	>=1% and <50%	32	2	0	4
CM227	NIVO	>=1% and <50%	34	2	1	4
CM227	NIVO	>=1% and <50%	36	2	0	3
CM227	NIVO	>=1% and <50%	38	2	0	3
CM227	NIVO	>=1% and <50%	40	2	0	3
CM227	NIVO	>=1% and <50%	42	2	0	3
CM227	NIVO	>=1% and <50%	44	2	0	3
CM227	NIVO	>=1% and <50%	46	2	0	3
CM227	NIVO	>=1% and <50%	48	2	0	3
CM227	NIVO	>=1% and <50%	50	2	0	3
CM227	NIVO	>=1% and <50%	52	2	0	3
CM227	NIVO	>=1% and <50%	54	2	0	3
CM227	NIVO	>=1% and <50%	56	2	0	3
CM227	NIVO	>=1% and <50%	58	2	0	3
CM227	NIVO	>=1% and <50%	60	2	0	3
CM227	NIVO	>=1% and <50%	62	2	0	3
CM227	NIVO+IPI	>=1% and <50%	2	2	0	191
CM227	NIVO+IPI	>=1% and <50%	4	2	56	173
CM227	NIVO+IPI	>=1% and <50%	6	2	29	101
CM227	NIVO+IPI	>=1% and <50%	8	2	13	65
CM227	NIVO+IPI	>=1% and <50%	10	2	9	52
CM227	NIVO+IPI	>=1% and <50%	12	2	6	43
CM227	NIVO+IPI	>=1% and <50%	14	2	5	36
CM227	NIVO+IPI	>=1% and <50%	16	2	4	31
CM227	NIVO+IPI	>=1% and <50%	18	2	3	27
CM227	NIVO+IPI	>=1% and <50%	20	2	2	23
CM227	NIVO+IPI	>=1% and <50%	22	2	2	21
CM227	NIVO+IPI	>=1% and <50%	24	2	1	19
CM227	NIVO+IPI	>=1% and <50%	26	2	2	18
CM227	NIVO+IPI	>=1% and <50%	28	2	0	16
CM227	NIVO+IPI	>=1% and <50%	30	2	1	16
CM227	NIVO+IPI	>=1% and <50%	32	2	0	15
CM227	NIVO+IPI	>=1% and <50%	34	2	0	15
CM227	NIVO+IPI	>=1% and <50%	36	2	2	15
CM227	NIVO+IPI	>=1% and <50%	38	2	0	13
CM227	NIVO+IPI	>=1% and <50%	40	2	1	13
CM227	NIVO+IPI	>=1% and <50%	42	2	1	12
CM227	NIVO+IPI	>=1% and <50%	44	2	0	11

CM227	NIVO+IPI	>=1% and <50%	46	2	0	11
CM227	NIVO+IPI	>=1% and <50%	48	2	0	11
CM227	NIVO+IPI	>=1% and <50%	50	2	2	10
CM227	NIVO+IPI	>=1% and <50%	52	2	0	8
CM227	NIVO+IPI	>=1% and <50%	54	2	0	8
CM227	NIVO+IPI	>=1% and <50%	56	2	0	8
CM227	NIVO+IPI	>=1% and <50%	58	2	1	8
CM227	NIVO+IPI	>=1% and <50%	60	2	0	7
CM227	NIVO+IPI	>=1% and <50%	62	2	1	7
CM227	NIVO+IPI	>=1% and <50%	64	2	0	6
CM227	NIVO+IPI	>=1% and <50%	66	2	0	5
CM227	NIVO+IPI	>=1% and <50%	68	2	0	3
CM227	NIVO+IPI	>=1% and <50%	70	2	0	2
CM227	NIVO+IPI	>=1% and <50%	72	2	0	1
CM227	NIVO+IPI	>=1% and <50%	74	2	0	1
CM227	NIVO+IPI	all	2	2	0	583
CM227	NIVO+IPI	all	4	2	162	530
CM227	NIVO+IPI	all	6	2	74	317
CM227	NIVO+IPI	all	8	2	35	226
CM227	NIVO+IPI	all	10	2	22	191
CM227	NIVO+IPI	all	12	2	19	169
CM227	NIVO+IPI	all	14	2	15	150
CM227	NIVO+IPI	all	16	2	10	135
CM227	NIVO+IPI	all	18	2	7	125
CM227	NIVO+IPI	all	20	2	6	118
CM227	NIVO+IPI	all	22	2	14	112
CM227	NIVO+IPI	all	24	2	4	98
CM227	NIVO+IPI	all	26	2	3	91
CM227	NIVO+IPI	all	28	2	3	88
CM227	NIVO+IPI	all	30	2	5	85
CM227	NIVO+IPI	all	32	2	0	76
CM227	NIVO+IPI	all	34	2	3	76
CM227	NIVO+IPI	all	36	2	2	73
CM227	NIVO+IPI	all	38	2	4	69
CM227	NIVO+IPI	all	40	2	3	65
CM227	NIVO+IPI	all	42	2	1	61
CM227	NIVO+IPI	all	44	2	4	60
CM227	NIVO+IPI	all	46	2	1	55
CM227	NIVO+IPI	all	48	2	2	53
CM227	NIVO+IPI	all	50	2	2	51
CM227	NIVO+IPI	all	52	2	0	49
CM227	NIVO+IPI	all	54	2	0	49
CM227	NIVO+IPI	all	56	2	0	49
CM227	NIVO+IPI	all	58	2	3	49

CM227	NIVO+IPI	all	60	2	2	46
CM227	NIVO+IPI	all	62	2	3	37
CM227	NIVO+IPI	all	64	2	1	28
CM227	NIVO+IPI	all	66	2	1	21
CM227	NIVO+IPI	all	68	2	0	12
CM227	NIVO+IPI	all	70	2	0	9
CM227	NIVO+IPI	all	72	2	0	6
CM227	NIVO+IPI	all	74	2	0	1
CM227	NIVO	>=50%	2	2	0	214
CM227	NIVO	>=50%	4	2	62	196
CM227	NIVO	>=50%	6	2	20	117
CM227	NIVO	>=50%	8	2	14	93
CM227	NIVO	>=50%	10	2	7	79
CM227	NIVO	>=50%	12	2	12	72
CM227	NIVO	>=50%	14	2	3	57
CM227	NIVO	>=50%	16	2	4	54
CM227	NIVO	>=50%	18	2	4	50
CM227	NIVO	>=50%	20	2	6	44
CM227	NIVO	>=50%	22	2	7	38
CM227	NIVO	>=50%	24	2	0	31
CM227	NIVO	>=50%	26	2	2	31
CM227	NIVO	>=50%	28	2	1	29
CM227	NIVO	>=50%	30	2	3	28
CM227	NIVO	>=50%	32	2	0	23
CM227	NIVO	>=50%	34	2	0	23
CM227	NIVO	>=50%	36	2	1	23
CM227	NIVO	>=50%	38	2	0	22
CM227	NIVO	>=50%	40	2	0	22
CM227	NIVO	>=50%	42	2	0	22
CM227	NIVO	>=50%	44	2	0	22
CM227	NIVO	>=50%	46	2	1	22
CM227	NIVO	>=50%	48	2	0	21
CM227	NIVO	>=50%	50	2	1	21
CM227	NIVO	>=50%	52	2	0	20
CM227	NIVO	>=50%	54	2	0	20
CM227	NIVO	>=50%	56	2	2	20
CM227	NIVO	>=50%	58	2	0	18
CM227	NIVO	>=50%	60	2	0	18
CM227	NIVO	>=50%	62	2	1	15
CM227	NIVO	>=50%	64	2	0	11
CM227	NIVO	>=50%	66	2	0	7
CM227	NIVO	>=50%	68	2	0	4
CM227	NIVO	>=50%	70	2	0	1
CM227	NIVO+IPI	>=50%	2	2	0	205

CM227	NIVO+IPI	>=50%	4	2	61	193
CM227	NIVO+IPI	>=50%	6	2	14	121
CM227	NIVO+IPI	>=50%	8	2	14	95
CM227	NIVO+IPI	>=50%	10	2	6	81
CM227	NIVO+IPI	>=50%	12	2	6	75
CM227	NIVO+IPI	>=50%	14	2	3	69
CM227	NIVO+IPI	>=50%	16	2	4	66
CM227	NIVO+IPI	>=50%	18	2	3	62
CM227	NIVO+IPI	>=50%	20	2	1	59
CM227	NIVO+IPI	>=50%	22	2	6	58
CM227	NIVO+IPI	>=50%	24	2	1	52
CM227	NIVO+IPI	>=50%	26	2	2	48
CM227	NIVO+IPI	>=50%	28	2	0	46
CM227	NIVO+IPI	>=50%	30	2	3	46
CM227	NIVO+IPI	>=50%	32	2	0	40
CM227	NIVO+IPI	>=50%	34	2	1	40
CM227	NIVO+IPI	>=50%	36	2	3	39
CM227	NIVO+IPI	>=50%	38	2	2	36
CM227	NIVO+IPI	>=50%	40	2	0	34
CM227	NIVO+IPI	>=50%	42	2	1	34
CM227	NIVO+IPI	>=50%	44	2	2	33
CM227	NIVO+IPI	>=50%	46	2	2	31
CM227	NIVO+IPI	>=50%	48	2	1	29
CM227	NIVO+IPI	>=50%	50	2	1	28
CM227	NIVO+IPI	>=50%	52	2	0	27
CM227	NIVO+IPI	>=50%	54	2	0	27
CM227	NIVO+IPI	>=50%	56	2	0	27
CM227	NIVO+IPI	>=50%	58	2	0	27
CM227	NIVO+IPI	>=50%	60	2	0	27
CM227	NIVO+IPI	>=50%	62	2	2	21
CM227	NIVO+IPI	>=50%	64	2	1	16
CM227	NIVO+IPI	>=50%	66	2	0	12
CM227	NIVO+IPI	>=50%	68	2	0	6
CM227	NIVO+IPI	>=50%	70	2	0	4
CM227	NIVO	>=1%	2	2	0	396
CM227	NIVO	>=1%	4	2	128	360
CM227	NIVO	>=1%	6	2	40	199
CM227	NIVO	>=1%	8	2	30	135
CM227	NIVO	>=1%	10	2	15	105
CM227	NIVO	>=1%	12	2	17	90
CM227	NIVO	>=1%	14	2	6	73
CM227	NIVO	>=1%	16	2	7	67
CM227	NIVO	>=1%	18	2	5	60
CM227	NIVO	>=1%	20	2	8	55

CM227	NIVO	>=1%	22	2	9	47
CM227	NIVO	>=1%	24	2	0	38
CM227	NIVO	>=1%	26	2	1	38
CM227	NIVO	>=1%	28	2	2	37
CM227	NIVO	>=1%	30	2	2	35
CM227	NIVO	>=1%	32	2	2	31
CM227	NIVO	>=1%	34	2	1	29
CM227	NIVO	>=1%	36	2	1	28
CM227	NIVO	>=1%	38	2	0	27
CM227	NIVO	>=1%	40	2	0	27
CM227	NIVO	>=1%	42	2	1	27
CM227	NIVO	>=1%	44	2	0	26
CM227	NIVO	>=1%	46	2	0	26
CM227	NIVO	>=1%	48	2	1	26
CM227	NIVO	>=1%	50	2	1	25
CM227	NIVO	>=1%	52	2	0	24
CM227	NIVO	>=1%	54	2	0	24
CM227	NIVO	>=1%	56	2	1	24
CM227	NIVO	>=1%	58	2	1	23
CM227	NIVO	>=1%	60	2	0	22
CM227	NIVO	>=1%	62	2	1	18
CM227	NIVO	>=1%	64	2	0	13
CM227	NIVO	>=1%	66	2	0	9
CM227	NIVO	>=1%	68	2	0	4
CM227	NIVO	>=1%	70	2	0	2
CM227	NIVO+IPI	>=1%	2	2	0	396
CM227	NIVO+IPI	>=1%	4	2	116	364
CM227	NIVO+IPI	>=1%	6	2	46	222
CM227	NIVO+IPI	>=1%	8	2	28	160
CM227	NIVO+IPI	>=1%	10	2	15	132
CM227	NIVO+IPI	>=1%	12	2	12	117
CM227	NIVO+IPI	>=1%	14	2	7	105
CM227	NIVO+IPI	>=1%	16	2	9	98
CM227	NIVO+IPI	>=1%	18	2	5	89
CM227	NIVO+IPI	>=1%	20	2	5	84
CM227	NIVO+IPI	>=1%	22	2	6	79
CM227	NIVO+IPI	>=1%	24	2	3	73
CM227	NIVO+IPI	>=1%	26	2	4	67
CM227	NIVO+IPI	>=1%	28	2	1	63
CM227	NIVO+IPI	>=1%	30	2	4	62
CM227	NIVO+IPI	>=1%	32	2	1	56
CM227	NIVO+IPI	>=1%	34	2	1	55
CM227	NIVO+IPI	>=1%	36	2	4	54
CM227	NIVO+IPI	>=1%	38	2	1	50

CM227	NIVO+IPI	>=1%	40	2	1	49
CM227	NIVO+IPI	>=1%	42	2	2	48
CM227	NIVO+IPI	>=1%	44	2	3	46
CM227	NIVO+IPI	>=1%	46	2	2	43
CM227	NIVO+IPI	>=1%	48	2	2	40
CM227	NIVO+IPI	>=1%	50	2	1	38
CM227	NIVO+IPI	>=1%	52	2	0	37
CM227	NIVO+IPI	>=1%	54	2	0	37
CM227	NIVO+IPI	>=1%	56	2	1	37
CM227	NIVO+IPI	>=1%	58	2	2	36
CM227	NIVO+IPI	>=1%	60	2	0	34
CM227	NIVO+IPI	>=1%	62	2	1	28
CM227	NIVO+IPI	>=1%	64	2	2	23
CM227	NIVO+IPI	>=1%	66	2	0	18
CM227	NIVO+IPI	>=1%	68	2	0	9
CM227	NIVO+IPI	>=1%	70	2	0	7
CM227	NIVO+IPI	>=1%	72	2	0	4
CM227	NIVO+IPI	>=1%	74	2	0	1
CM227	NIVO+chemo	<1%	2	2	0	177
CM227	NIVO+chemo	<1%	4	2	24	168
CM227	NIVO+chemo	<1%	6	2	14	135
CM227	NIVO+chemo	<1%	8	2	31	73
CM227	NIVO+chemo	<1%	10	2	6	42
CM227	NIVO+chemo	<1%	12	2	11	36
CM227	NIVO+chemo	<1%	14	2	1	25
CM227	NIVO+chemo	<1%	16	2	3	24
CM227	NIVO+chemo	<1%	18	2	3	21
CM227	NIVO+chemo	<1%	20	2	5	18
CM227	NIVO+chemo	<1%	22	2	0	12
CM227	NIVO+chemo	<1%	24	2	1	12
CM227	NIVO+chemo	<1%	26	2	2	11
CM227	NIVO+chemo	<1%	28	2	0	9
CM227	NIVO+chemo	<1%	30	2	0	9
CM227	NIVO+chemo	<1%	32	2	1	9
CM227	NIVO+chemo	<1%	34	2	1	8
CM227	NIVO+chemo	<1%	36	2	0	7
CM227	NIVO+chemo	<1%	38	2	0	7
CM227	NIVO+chemo	<1%	40	2	0	7
CM227	NIVO+chemo	<1%	42	2	1	7
CM227	NIVO+chemo	<1%	44	2	0	6
CM227	NIVO+chemo	<1%	46	2	0	6
CM227	NIVO+chemo	<1%	48	2	0	6
CM227	NIVO+chemo	<1%	50	2	0	6
CM227	NIVO+chemo	<1%	52	2	0	6

CM227	NIVO+chemo	<1%	54	2	0	6
CM227	NIVO+chemo	<1%	56	2	0	6
CM227	NIVO+chemo	<1%	58	2	0	5
CM227	NIVO+chemo	<1%	60	2	0	5
CM227	NIVO+chemo	<1%	62	2	0	3
CM227	NIVO+chemo	<1%	64	2	0	3
CM227	NIVO+chemo	<1%	66	2	0	3
CM227	NIVO+chemo	<1%	68	2	0	0
CM227	NIVO+IPI	<1%	2	2	0	187
CM227	NIVO+IPI	<1%	4	2	48	165
CM227	NIVO+IPI	<1%	6	2	20	95
CM227	NIVO+IPI	<1%	8	2	13	66
CM227	NIVO+IPI	<1%	10	2	8	53
CM227	NIVO+IPI	<1%	12	2	6	45
CM227	NIVO+IPI	<1%	14	2	3	39
CM227	NIVO+IPI	<1%	16	2	5	36
CM227	NIVO+IPI	<1%	18	2	0	31
CM227	NIVO+IPI	<1%	20	2	3	31
CM227	NIVO+IPI	<1%	22	2	4	28
CM227	NIVO+IPI	<1%	24	2	2	24
CM227	NIVO+IPI	<1%	26	2	1	22
CM227	NIVO+IPI	<1%	28	2	1	21
CM227	NIVO+IPI	<1%	30	2	1	20
CM227	NIVO+IPI	<1%	32	2	0	19
CM227	NIVO+IPI	<1%	34	2	1	19
CM227	NIVO+IPI	<1%	36	2	1	18
CM227	NIVO+IPI	<1%	38	2	1	16
CM227	NIVO+IPI	<1%	40	2	0	14
CM227	NIVO+IPI	<1%	42	2	1	14
CM227	NIVO+IPI	<1%	44	2	0	13
CM227	NIVO+IPI	<1%	46	2	0	13
CM227	NIVO+IPI	<1%	48	2	0	13
CM227	NIVO+IPI	<1%	50	2	0	13
CM227	NIVO+IPI	<1%	52	2	0	13
CM227	NIVO+IPI	<1%	54	2	0	13
CM227	NIVO+IPI	<1%	56	2	1	13
CM227	NIVO+IPI	<1%	58	2	1	11
CM227	NIVO+IPI	<1%	60	2	0	10
CM227	NIVO+IPI	<1%	62	2	1	9
CM227	NIVO+IPI	<1%	64	2	1	6
CM227	NIVO+IPI	<1%	66	2	1	3
CM227	NIVO+IPI	<1%	68	2	0	2
CM227	NIVO+IPI	<1%	70	2	0	2
IM110	ate	>=1%	2	2	14	277

IM110	ate	>=1%	4	2	67	199
IM110	ate	>=1%	6	2	12	132
IM110	ate	>=1%	8	2	24	120
IM110	ate	>=1%	10	2	15	96
IM110	ate	>=1%	12	2	9	81
IM110	ate	>=1%	14	2	8	72
IM110	ate	>=1%	16	2	6	64
IM110	ate	>=1%	18	2	6	58
IM110	ate	>=1%	20	2	4	52
IM110	ate	>=1%	22	2	3	48
IM110	ate	>=1%	24	2	3	45
IM110	ate	>=1%	26	2	4	42
IM110	ate	>=1%	28	2	0	31
IM110	ate	>=1%	30	2	2	24
IM110	ate	>=1%	32	2	0	21
IM110	ate	>=1%	34	2	1	16
IM110	ate	>=1%	36	2	0	12
IM110	ate	>=1%	38	2	0	8
IM110	ate	>=1%	40	2	0	7
IM110	ate	>=1%	42	2	0	7
IM110	ate	>=1%	44	2	1	3
IM110	ate	>=1%	46	2	0	2
IM110	ate	>=1%	48	2	1	2
IM110	ate	>=1%	50	2	0	1
IM110	ate	>=1%	52	2	1	1
IM110	ate	>=50%	2	2	6	107
IM110	ate	>=50%	4	2	22	83
IM110	ate	>=50%	6	2	4	60
IM110	ate	>=50%	8	2	8	56
IM110	ate	>=50%	10	2	9	48
IM110	ate	>=50%	12	2	3	39
IM110	ate	>=50%	14	2	4	36
IM110	ate	>=50%	16	2	1	32
IM110	ate	>=50%	18	2	1	31
IM110	ate	>=50%	20	2	2	30
IM110	ate	>=50%	22	2	2	28
IM110	ate	>=50%	24	2	1	26
IM110	ate	>=50%	26	2	3	25
IM110	ate	>=50%	28	2	0	21
IM110	ate	>=50%	30	2	1	15
IM110	ate	>=50%	32	2	0	14
IM110	ate	>=50%	34	2	0	12
IM110	ate	>=50%	36	2	0	11
IM110	ate	>=50%	38	2	0	8

IM110	ate	>=50%	40	2	0	7
IM110	ate	>=50%	42	2	0	7
IM110	ate	>=50%	44	2	1	3
IM110	ate	>=50%	46	2	0	2
IM110	ate	>=50%	48	2	1	2
IM110	ate	>=50%	50	2	0	1
IM110	ate	>=50%	52	2	1	1
IM130	ate+chemo	all	2	2	13	451
IM130	ate+chemo	all	4	2	70	409
IM130	ate+chemo	all	6	2	64	319
IM130	ate+chemo	all	8	2	54	242
IM130	ate+chemo	all	10	2	50	184
IM130	ate+chemo	all	12	2	17	131
IM130	ate+chemo	all	14	2	17	111
IM130	ate+chemo	all	16	2	10	87
IM130	ate+chemo	all	18	2	4	64
IM130	ate+chemo	all	20	2	5	41
IM130	ate+chemo	all	22	2	5	29
IM130	ate+chemo	all	24	2	4	18
IM130	ate+chemo	all	26	2	1	7
IM130	ate+chemo	all	28	2	0	0
IM132	ate+car/cis+peme	>=1% and <50%	2	2	1	63
IM132	ate+car/cis+peme	>=1% and <50%	4	2	11	55
IM132	ate+car/cis+peme	>=1% and <50%	6	2	10	44
IM132	ate+car/cis+peme	>=1% and <50%	8	2	6	29
IM132	ate+car/cis+peme	>=1% and <50%	10	2	7	23
IM132	ate+car/cis+peme	>=1% and <50%	12	2	1	15
IM132	ate+car/cis+peme	>=1% and <50%	14	2	3	14
IM132	ate+car/cis+peme	>=1% and <50%	16	2	2	8
IM132	ate+car/cis+peme	>=1% and <50%	18	2	2	5
IM132	ate+car/cis+peme	>=1% and <50%	20	2	0	3
IM132	ate+car/cis+peme	all	2	2	8	292
IM132	ate+car/cis+peme	all	4	2	45	260
IM132	ate+car/cis+peme	all	6	2	32	202
IM132	ate+car/cis+peme	all	8	2	37	163
IM132	ate+car/cis+peme	all	10	2	24	126
IM132	ate+car/cis+peme	all	12	2	10	102
IM132	ate+car/cis+peme	all	14	2	15	88
IM132	ate+car/cis+peme	all	16	2	5	48
IM132	ate+car/cis+peme	all	18	2	5	31
IM132	ate+car/cis+peme	all	20	2	1	11
IM132	ate+car/cis+peme	all	22	2	0	2
IM132	ate+car/cis+peme	>=50%	2	2	0	25
IM132	ate+car/cis+peme	>=50%	4	2	3	24

IM132	ate+car/cis+peme	>=50%	6	2	1	18
IM132	ate+car/cis+peme	>=50%	8	2	2	17
IM132	ate+car/cis+peme	>=50%	10	2	3	15
IM132	ate+car/cis+peme	>=50%	12	2	1	11
IM132	ate+car/cis+peme	>=50%	14	2	1	10
IM132	ate+car/cis+peme	>=50%	16	2	0	7
IM132	ate+car/cis+peme	>=50%	18	2	0	5
IM132	ate+car/cis+peme	<1%	2	2	2	88
IM132	ate+car/cis+peme	<1%	4	2	15	76
IM132	ate+car/cis+peme	<1%	6	2	10	60
IM132	ate+car/cis+peme	<1%	8	2	6	48
IM132	ate+car/cis+peme	<1%	10	2	6	42
IM132	ate+car/cis+peme	<1%	12	2	5	36
IM132	ate+car/cis+peme	<1%	14	2	6	26
IM132	ate+car/cis+peme	<1%	16	2	2	13
IM132	ate+car/cis+peme	<1%	18	2	0	10
IM132	ate+car/cis+peme	<1%	20	2	2	5
IM132	ate+car/cis+peme	<1%	22	2	0	2
IM150	ate+beva+car+pac	>=1% and <50%	2	2	5	119
IM150	ate+beva+car+pac	>=1% and <50%	4	2	7	105
IM150	ate+beva+car+pac	>=1% and <50%	6	2	9	98
IM150	ate+beva+car+pac	>=1% and <50%	8	2	17	85
IM150	ate+beva+car+pac	>=1% and <50%	10	2	16	64
IM150	ate+beva+car+pac	>=1% and <50%	12	2	6	44
IM150	ate+beva+car+pac	>=1% and <50%	14	2	3	28
IM150	ate+beva+car+pac	>=1% and <50%	16	2	4	20
IM150	ate+beva+car+pac	>=1% and <50%	18	2	1	14
IM150	ate+beva+car+pac	>=1% and <50%	20	2	1	8
IM150	ate+beva+car+pac	>=1% and <50%	22	2	0	4
IM150	ate+beva+car+pac	all	2	2	14	356
IM150	ate+beva+car+pac	all	4	2	35	310
IM150	ate+beva+car+pac	all	6	2	32	270
IM150	ate+beva+car+pac	all	8	2	49	221
IM150	ate+beva+car+pac	all	10	2	31	172
IM150	ate+beva+car+pac	all	12	2	23	110
IM150	ate+beva+car+pac	all	14	2	10	82
IM150	ate+beva+car+pac	all	16	2	7	56
IM150	ate+beva+car+pac	all	18	2	7	39
IM150	ate+beva+car+pac	all	20	2	4	25
IM150	ate+beva+car+pac	all	22	2	1	13
IM150	ate+beva+car+pac	all	24	2	2	3
IM150	ate+beva+car+pac	all	26	2	0	1
IM150	ate+beva+car+pac	>=50%	2	2	3	71
IM150	ate+beva+car+pac	>=50%	4	2	7	60

IM150	ate+beva+car+pac	>=50%	6	2	4	53
IM150	ate+beva+car+pac	>=50%	8	2	2	49
IM150	ate+beva+car+pac	>=50%	10	2	7	47
IM150	ate+beva+car+pac	>=50%	12	2	1	36
IM150	ate+beva+car+pac	>=50%	14	2	7	27
IM150	ate+beva+car+pac	>=50%	16	2	1	18
IM150	ate+beva+car+pac	>=50%	18	2	1	16
IM150	ate+beva+car+pac	>=50%	20	2	0	13
IM150	ate+beva+car+pac	>=50%	22	2	0	8
IM150	ate+beva+car+pac	>=50%	24	2	1	3
IM150	ate+beva+car+pac	>=50%	26	2	0	2
IM150	ate+beva+car+pac	<1%	2	2	7	166
IM150	ate+beva+car+pac	<1%	4	2	17	146
IM150	ate+beva+car+pac	<1%	6	2	18	129
IM150	ate+beva+car+pac	<1%	8	2	27	94
IM150	ate+beva+car+pac	<1%	10	2	16	66
IM150	ate+beva+car+pac	<1%	12	2	8	44
IM150	ate+beva+car+pac	<1%	14	2	5	32
IM150	ate+beva+car+pac	<1%	16	2	4	18
IM150	ate+beva+car+pac	<1%	18	2	0	12
IM150	ate+beva+car+pac	<1%	20	2	1	6
IM150	ate+beva+car+pac	<1%	22	2	1	4
KN042	pem	>=1%	2	2	37	637
KN042	pem	>=1%	4	2	175	587
KN042	pem	>=1%	6	2	90	397
KN042	pem	>=1%	8	2	53	295
KN042	pem	>=1%	10	2	37	237
KN042	pem	>=1%	12	2	38	194
KN042	pem	>=1%	14	2	12	152
KN042	pem	>=1%	16	2	16	131
KN042	pem	>=1%	18	2	8	102
KN042	pem	>=1%	20	2	8	81
KN042	pem	>=1%	22	2	2	65
KN042	pem	>=1%	24	2	9	58
KN042	pem	>=1%	26	2	3	42
KN042	pem	>=1%	28	2	1	29
KN042	pem	>=1%	30	2	0	19
KN042	pem	>=1%	32	2	1	8
KN042	pem	>=1%	34	2	0	3
KN042	pem	>=1%	36	2	0	0
KN042	pem	>=50%	2	2	22	299
KN042	pem	>=50%	4	2	65	273
KN042	pem	>=50%	6	2	36	203
KN042	pem	>=50%	8	2	23	163

KN042	pem	>=50%	10	2	16	137
KN042	pem	>=50%	12	2	14	117
KN042	pem	>=50%	14	2	11	100
KN042	pem	>=50%	16	2	9	81
KN042	pem	>=50%	18	2	3	61
KN042	pem	>=50%	20	2	3	52
KN042	pem	>=50%	22	2	1	44
KN042	pem	>=50%	24	2	6	38
KN042	pem	>=50%	26	2	2	27
KN042	pem	>=50%	28	2	1	19
KN042	pem	>=50%	30	2	0	11
KN042	pem	>=50%	32	2	1	6
KN042	pem	>=50%	34	2	0	1
KN042	pem	>=50%	36	2	0	0
KN189	pem+chemo	>=1% and <50%	2	2	0	128
KN189	pem+chemo	>=1% and <50%	4	2	13	126
KN189	pem+chemo	>=1% and <50%	6	2	9	111
KN189	pem+chemo	>=1% and <50%	8	2	13	100
KN189	pem+chemo	>=1% and <50%	10	2	14	85
KN189	pem+chemo	>=1% and <50%	12	2	13	69
KN189	pem+chemo	>=1% and <50%	14	2	5	54
KN189	pem+chemo	>=1% and <50%	16	2	5	49
KN189	pem+chemo	>=1% and <50%	18	2	4	44
KN189	pem+chemo	>=1% and <50%	20	2	4	40
KN189	pem+chemo	>=1% and <50%	22	2	2	35
KN189	pem+chemo	>=1% and <50%	24	2	4	33
KN189	pem+chemo	>=1% and <50%	26	2	4	29
KN189	pem+chemo	>=1% and <50%	28	2	5	25
KN189	pem+chemo	>=1% and <50%	30	2	1	20
KN189	pem+chemo	>=1% and <50%	32	2	1	18
KN189	pem+chemo	>=1% and <50%	34	2	1	17
KN189	pem+chemo	>=1% and <50%	36	2	1	16
KN189	pem+chemo	>=1% and <50%	38	2	2	15
KN189	pem+chemo	>=1% and <50%	40	2	2	13
KN189	pem+chemo	>=1% and <50%	42	2	2	11
KN189	pem+chemo	>=1% and <50%	44	2	1	9
KN189	pem+chemo	>=1% and <50%	46	2	0	8
KN189	pem+chemo	>=1% and <50%	48	2	0	8
KN189	pem+chemo	>=1% and <50%	50	2	0	8
KN189	pem+chemo	>=1% and <50%	52	2	0	8
KN189	pem+chemo	>=1% and <50%	54	2	0	8
KN189	pem+chemo	>=1% and <50%	56	2	1	8
KN189	pem+chemo	>=1% and <50%	58	2	0	6
KN189	pem+chemo	>=1% and <50%	60	2	0	6

KN189	pem+chemo	>=1% and <50%	62	2	0	6
KN189	pem+chemo	>=1% and <50%	64	2	0	6
KN189	pem+chemo	>=1% and <50%	66	2	1	5
KN189	pem+chemo	>=1% and <50%	68	2	1	4
KN189	pem+chemo	>=1% and <50%	70	2	0	2
KN189	pem+chemo	all	2	2	0	410
KN189	pem+chemo	all	4	2	38	402
KN189	pem+chemo	all	6	2	41	357
KN189	pem+chemo	all	8	2	48	309
KN189	pem+chemo	all	10	2	45	253
KN189	pem+chemo	all	12	2	36	201
KN189	pem+chemo	all	14	2	17	158
KN189	pem+chemo	all	16	2	18	141
KN189	pem+chemo	all	18	2	6	123
KN189	pem+chemo	all	20	2	16	116
KN189	pem+chemo	all	22	2	5	100
KN189	pem+chemo	all	24	2	8	95
KN189	pem+chemo	all	26	2	6	87
KN189	pem+chemo	all	28	2	12	81
KN189	pem+chemo	all	30	2	3	69
KN189	pem+chemo	all	32	2	5	66
KN189	pem+chemo	all	34	2	9	60
KN189	pem+chemo	all	36	2	3	51
KN189	pem+chemo	all	38	2	2	48
KN189	pem+chemo	all	40	2	3	46
KN189	pem+chemo	all	42	2	5	43
KN189	pem+chemo	all	44	2	2	38
KN189	pem+chemo	all	46	2	0	36
KN189	pem+chemo	all	48	2	0	36
KN189	pem+chemo	all	50	2	0	36
KN189	pem+chemo	all	52	2	2	35
KN189	pem+chemo	all	54	2	0	31
KN189	pem+chemo	all	56	2	3	29
KN189	pem+chemo	all	58	2	0	25
KN189	pem+chemo	all	60	2	1	23
KN189	pem+chemo	all	62	2	3	21
KN189	pem+chemo	all	64	2	0	17
KN189	pem+chemo	all	66	2	1	15
KN189	pem+chemo	all	68	2	1	12
KN189	pem+chemo	all	70	2	0	9
KN189	pem+chemo	all	72	2	0	8
KN189	pem+chemo	>=1%	2	2	0	260
KN189	pem+chemo	>=1%	4	2	24	257
KN189	pem+chemo	>=1%	6	2	16	229

KN189	pem+chemo	>=1%	8	2	27	209
KN189	pem+chemo	>=1%	10	2	29	178
KN189	pem+chemo	>=1%	12	2	25	145
KN189	pem+chemo	>=1%	14	2	9	117
KN189	pem+chemo	>=1%	16	2	12	108
KN189	pem+chemo	>=1%	18	2	4	96
KN189	pem+chemo	>=1%	20	2	10	91
KN189	pem+chemo	>=1%	22	2	7	81
KN189	pem+chemo	>=1%	24	2	5	74
KN189	pem+chemo	>=1%	26	2	6	69
KN189	pem+chemo	>=1%	28	2	9	63
KN189	pem+chemo	>=1%	30	2	3	53
KN189	pem+chemo	>=1%	32	2	3	50
KN189	pem+chemo	>=1%	34	2	4	46
KN189	pem+chemo	>=1%	36	2	3	41
KN189	pem+chemo	>=1%	38	2	3	38
KN189	pem+chemo	>=1%	40	2	3	35
KN189	pem+chemo	>=1%	42	2	4	32
KN189	pem+chemo	>=1%	44	2	2	28
KN189	pem+chemo	>=1%	46	2	0	26
KN189	pem+chemo	>=1%	48	2	0	26
KN189	pem+chemo	>=1%	50	2	0	26
KN189	pem+chemo	>=1%	52	2	0	25
KN189	pem+chemo	>=1%	54	2	0	24
KN189	pem+chemo	>=1%	56	2	1	22
KN189	pem+chemo	>=1%	58	2	2	20
KN189	pem+chemo	>=1%	60	2	0	17
KN189	pem+chemo	>=1%	62	2	1	16
KN189	pem+chemo	>=1%	64	2	0	14
KN189	pem+chemo	>=1%	66	2	1	13
KN189	pem+chemo	>=1%	68	2	1	11
KN189	pem+chemo	>=1%	70	2	0	9
KN189	pem+chemo	>=1%	72	2	0	8
KN189	pem+chemo	>=50%	2	2	0	132
KN189	pem+chemo	>=50%	4	2	11	131
KN189	pem+chemo	>=50%	6	2	8	118
KN189	pem+chemo	>=50%	8	2	13	108
KN189	pem+chemo	>=50%	10	2	14	93
KN189	pem+chemo	>=50%	12	2	13	77
KN189	pem+chemo	>=50%	14	2	4	63
KN189	pem+chemo	>=50%	16	2	6	59
KN189	pem+chemo	>=50%	18	2	2	53
KN189	pem+chemo	>=50%	20	2	7	51
KN189	pem+chemo	>=50%	22	2	1	44

KN189	pem+chemo	>=50%	24	2	3	43
KN189	pem+chemo	>=50%	26	2	3	40
KN189	pem+chemo	>=50%	28	2	2	37
KN189	pem+chemo	>=50%	30	2	3	34
KN189	pem+chemo	>=50%	32	2	4	30
KN189	pem+chemo	>=50%	34	2	1	25
KN189	pem+chemo	>=50%	36	2	0	23
KN189	pem+chemo	>=50%	38	2	2	23
KN189	pem+chemo	>=50%	40	2	1	21
KN189	pem+chemo	>=50%	42	2	2	20
KN189	pem+chemo	>=50%	44	2	0	18
KN189	pem+chemo	>=50%	46	2	0	18
KN189	pem+chemo	>=50%	48	2	1	18
KN189	pem+chemo	>=50%	50	2	0	17
KN189	pem+chemo	>=50%	52	2	0	16
KN189	pem+chemo	>=50%	54	2	0	15
KN189	pem+chemo	>=50%	56	2	1	14
KN189	pem+chemo	>=50%	58	2	0	12
KN189	pem+chemo	>=50%	60	2	0	11
KN189	pem+chemo	>=50%	62	2	2	10
KN189	pem+chemo	>=50%	64	2	0	8
KN189	pem+chemo	>=50%	66	2	0	7
KN189	pem+chemo	>=50%	68	2	0	6
KN189	pem+chemo	>=50%	70	2	0	5
KN189	pem+chemo	>=50%	72	2	0	5
KN189	pem+chemo	<1%	2	2	0	127
KN189	pem+chemo	<1%	4	2	13	125
KN189	pem+chemo	<1%	6	2	24	109
KN189	pem+chemo	<1%	8	2	20	82
KN189	pem+chemo	<1%	10	2	12	59
KN189	pem+chemo	<1%	12	2	9	44
KN189	pem+chemo	<1%	14	2	7	33
KN189	pem+chemo	<1%	16	2	2	26
KN189	pem+chemo	<1%	18	2	1	24
KN189	pem+chemo	<1%	20	2	5	23
KN189	pem+chemo	<1%	22	2	2	18
KN189	pem+chemo	<1%	24	2	1	16
KN189	pem+chemo	<1%	26	2	1	15
KN189	pem+chemo	<1%	28	2	2	14
KN189	pem+chemo	<1%	30	2	2	12
KN189	pem+chemo	<1%	32	2	3	10
KN189	pem+chemo	<1%	34	2	1	7
KN189	pem+chemo	<1%	36	2	0	6
KN189	pem+chemo	<1%	38	2	0	6

KN189	pem+chemo	<1%	40	2	1	6
KN189	pem+chemo	<1%	42	2	0	5
KN189	pem+chemo	<1%	44	2	0	5
KN189	pem+chemo	<1%	46	2	0	5
KN189	pem+chemo	<1%	48	2	1	5
KN189	pem+chemo	<1%	50	2	0	4
KN189	pem+chemo	<1%	52	2	0	4
KN189	pem+chemo	<1%	54	2	0	4
KN189	pem+chemo	<1%	56	2	0	4
KN189	pem+chemo	<1%	58	2	0	4
KN189	pem+chemo	<1%	60	2	1	4
KN189	pem+chemo	<1%	62	2	1	3
KN189	pem+chemo	<1%	64	2	0	2
KN598	pem	>=50%	2	2	21	284
KN598	pem	>=50%	4	2	49	237
KN598	pem	>=50%	6	2	28	177
KN598	pem	>=50%	8	2	15	148
KN598	pem	>=50%	10	2	16	128
KN598	pem	>=50%	12	2	10	109
KN598	pem	>=50%	14	2	10	97
KN598	pem	>=50%	16	2	3	79
KN598	pem	>=50%	18	2	6	65
KN598	pem	>=50%	20	2	5	43
KN598	pem	>=50%	22	2	3	26
KN598	pem	>=50%	24	2	0	16
KN598	pem	>=50%	26	2	1	11
KN598	pem	>=50%	28	2	0	6
KN598	pem	>=50%	30	2	0	3
KN598	pem	>=50%	32	2	0	1
KN598	pem+ipi	>=50%	2	2	21	284
KN598	pem+ipi	>=50%	4	2	50	229
KN598	pem+ipi	>=50%	6	2	26	162
KN598	pem+ipi	>=50%	8	2	19	133
KN598	pem+ipi	>=50%	10	2	9	114
KN598	pem+ipi	>=50%	12	2	10	105
KN598	pem+ipi	>=50%	14	2	14	95
KN598	pem+ipi	>=50%	16	2	9	73
KN598	pem+ipi	>=50%	18	2	2	53
KN598	pem+ipi	>=50%	20	2	5	36
KN598	pem+ipi	>=50%	22	2	1	23
KN598	pem+ipi	>=50%	24	2	1	16
KN598	pem+ipi	>=50%	26	2	0	9
KN598	pem+ipi	>=50%	28	2	0	4
KN598	pem+ipi	>=50%	30	2	0	0

Table S4 Dateset of OS used to fractional polynomial analysis

Study	Treatment	PD-L1	Time	Time Interval	No.Events	No.at Risk
CM9LA	NIVO+IPI+chemo	all	2	2	10	361
CM9LA	NIVO+IPI+chemo	all	4	2	26	341
CM9LA	NIVO+IPI+chemo	all	6	2	23	310
CM9LA	NIVO+IPI+chemo	all	8	2	20	287
CM9LA	NIVO+IPI+chemo	all	10	2	29	267
CM9LA	NIVO+IPI+chemo	all	12	2	14	238
CM9LA	NIVO+IPI+chemo	all	14	2	19	224
CM9LA	NIVO+IPI+chemo	all	16	2	23	204
CM9LA	NIVO+IPI+chemo	all	18	2	16	181
CM9LA	NIVO+IPI+chemo	all	20	2	11	165
CM9LA	NIVO+IPI+chemo	all	22	2	10	154
CM9LA	NIVO+IPI+chemo	all	24	2	11	144
CM9LA	NIVO+IPI+chemo	all	26	2	7	133
CM9LA	NIVO+IPI+chemo	all	28	2	9	110
CM9LA	NIVO+IPI+chemo	all	30	2	4	77
CM9LA	NIVO+IPI+chemo	all	32	2	1	50
CM9LA	NIVO+IPI+chemo	all	34	2	2	32
CM9LA	NIVO+IPI+chemo	all	36	2	2	18
CM9LA	NIVO+IPI+chemo	all	38	2	0	7
CM9LA	NIVO+IPI+chemo	all	40	2	0	2
CM9LA	NIVO+IPI+chemo	>=1%	2	2	4	204
CM9LA	NIVO+IPI+chemo	>=1%	4	2	14	193
CM9LA	NIVO+IPI+chemo	>=1%	6	2	15	176
CM9LA	NIVO+IPI+chemo	>=1%	8	2	11	161
CM9LA	NIVO+IPI+chemo	>=1%	10	2	11	150
CM9LA	NIVO+IPI+chemo	>=1%	12	2	8	139
CM9LA	NIVO+IPI+chemo	>=1%	14	2	14	131
CM9LA	NIVO+IPI+chemo	>=1%	16	2	13	117
CM9LA	NIVO+IPI+chemo	>=1%	18	2	12	104
CM9LA	NIVO+IPI+chemo	>=1%	20	2	1	92
CM9LA	NIVO+IPI+chemo	>=1%	22	2	7	91
CM9LA	NIVO+IPI+chemo	>=1%	24	2	3	84
CM9LA	NIVO+IPI+chemo	>=1%	26	2	5	81
CM9LA	NIVO+IPI+chemo	>=1%	28	2	4	67
CM9LA	NIVO+IPI+chemo	>=1%	30	2	1	51
CM9LA	NIVO+IPI+chemo	>=1%	32	2	2	35
CM9LA	NIVO+IPI+chemo	>=1%	34	2	2	23
CM9LA	NIVO+IPI+chemo	>=1%	36	2	2	13
CM9LA	NIVO+IPI+chemo	>=1%	38	2	0	5
CM9LA	NIVO+IPI+chemo	>=1%	40	2	0	2
CM9LA	NIVO+IPI+chemo	>=50%	2	2	0	76

CM9LA	NIVO+IPI+chemo	>=50%	4	2	5	72
CM9LA	NIVO+IPI+chemo	>=50%	6	2	7	66
CM9LA	NIVO+IPI+chemo	>=50%	8	2	1	59
CM9LA	NIVO+IPI+chemo	>=50%	10	2	3	58
CM9LA	NIVO+IPI+chemo	>=50%	12	2	1	54
CM9LA	NIVO+IPI+chemo	>=50%	14	2	8	53
CM9LA	NIVO+IPI+chemo	>=50%	16	2	4	45
CM9LA	NIVO+IPI+chemo	>=50%	18	2	6	41
CM9LA	NIVO+IPI+chemo	>=50%	20	2	1	35
CM9LA	NIVO+IPI+chemo	>=50%	22	2	1	34
CM9LA	NIVO+IPI+chemo	>=50%	24	2	2	33
CM9LA	NIVO+IPI+chemo	>=50%	26	2	0	31
CM9LA	NIVO+IPI+chemo	>=50%	28	2	1	27
CM9LA	NIVO+IPI+chemo	>=50%	30	2	2	21
CM9LA	NIVO+IPI+chemo	>=50%	32	2	0	12
CM9LA	NIVO+IPI+chemo	>=50%	34	2	1	8
CM9LA	NIVO+IPI+chemo	>=50%	36	2	0	5
CM9LA	NIVO+IPI+chemo	>=50%	38	2	0	2
CM9LA	NIVO+IPI+chemo	>=50%	40	2	0	1
CM9LA	NIVO+IPI+chemo	<1%	2	2	5	135
CM9LA	NIVO+IPI+chemo	<1%	4	2	9	127
CM9LA	NIVO+IPI+chemo	<1%	6	2	8	116
CM9LA	NIVO+IPI+chemo	<1%	8	2	8	107
CM9LA	NIVO+IPI+chemo	<1%	10	2	14	99
CM9LA	NIVO+IPI+chemo	<1%	12	2	2	85
CM9LA	NIVO+IPI+chemo	<1%	14	2	6	83
CM9LA	NIVO+IPI+chemo	<1%	16	2	8	77
CM9LA	NIVO+IPI+chemo	<1%	18	2	4	69
CM9LA	NIVO+IPI+chemo	<1%	20	2	5	65
CM9LA	NIVO+IPI+chemo	<1%	22	2	8	60
CM9LA	NIVO+IPI+chemo	<1%	24	2	4	52
CM9LA	NIVO+IPI+chemo	<1%	26	2	3	48
CM9LA	NIVO+IPI+chemo	<1%	28	2	5	36
CM9LA	NIVO+IPI+chemo	<1%	30	2	1	23
CM9LA	NIVO+IPI+chemo	<1%	32	2	1	13
CM9LA	NIVO+IPI+chemo	<1%	34	2	0	7
CM9LA	NIVO+IPI+chemo	<1%	36	2	0	5
CM9LA	NIVO+IPI+chemo	<1%	38	2	0	2
CM9LA	NIVO+IPI+chemo	<1%	40	2	0	0
CM227	NIVO	>=1% and <50%	2	2	0	182
CM227	NIVO	>=1% and <50%	4	2	19	175
CM227	NIVO	>=1% and <50%	6	2	18	149
CM227	NIVO	>=1% and <50%	8	2	10	130
CM227	NIVO	>=1% and <50%	10	2	12	120

CM227	NIVO	>=1% and <50%	12	2	8	108
CM227	NIVO	>=1% and <50%	14	2	12	93
CM227	NIVO	>=1% and <50%	16	2	5	81
CM227	NIVO	>=1% and <50%	18	2	9	76
CM227	NIVO	>=1% and <50%	20	2	6	67
CM227	NIVO	>=1% and <50%	22	2	5	61
CM227	NIVO	>=1% and <50%	24	2	7	56
CM227	NIVO	>=1% and <50%	26	2	3	49
CM227	NIVO	>=1% and <50%	28	2	0	46
CM227	NIVO	>=1% and <50%	30	2	8	46
CM227	NIVO	>=1% and <50%	32	2	2	35
CM227	NIVO	>=1% and <50%	34	2	0	33
CM227	NIVO	>=1% and <50%	36	2	2	33
CM227	NIVO	>=1% and <50%	38	2	1	31
CM227	NIVO	>=1% and <50%	40	2	2	30
CM227	NIVO	>=1% and <50%	42	2	1	28
CM227	NIVO	>=1% and <50%	44	2	0	27
CM227	NIVO	>=1% and <50%	46	2	3	27
CM227	NIVO	>=1% and <50%	48	2	2	24
CM227	NIVO	>=1% and <50%	50	2	1	22
CM227	NIVO	>=1% and <50%	52	2	1	21
CM227	NIVO	>=1% and <50%	54	2	0	20
CM227	NIVO	>=1% and <50%	56	2	2	20
CM227	NIVO	>=1% and <50%	58	2	1	18
CM227	NIVO	>=1% and <50%	60	2	1	17
CM227	NIVO	>=1% and <50%	62	2	1	16
CM227	NIVO	>=1% and <50%	64	2	0	14
CM227	NIVO	>=1% and <50%	66	2	0	13
CM227	NIVO	>=1% and <50%	68	2	0	10
CM227	NIVO	>=1% and <50%	70	2	0	7
CM227	NIVO	>=1% and <50%	72	2	0	5
CM227	NIVO+IPI	>=1% and <50%	2	2	0	191
CM227	NIVO+IPI	>=1% and <50%	4	2	18	189
CM227	NIVO+IPI	>=1% and <50%	6	2	8	169
CM227	NIVO+IPI	>=1% and <50%	8	2	19	139
CM227	NIVO+IPI	>=1% and <50%	10	2	9	120
CM227	NIVO+IPI	>=1% and <50%	12	2	12	111
CM227	NIVO+IPI	>=1% and <50%	14	2	8	99
CM227	NIVO+IPI	>=1% and <50%	16	2	6	91
CM227	NIVO+IPI	>=1% and <50%	18	2	5	85
CM227	NIVO+IPI	>=1% and <50%	20	2	9	80
CM227	NIVO+IPI	>=1% and <50%	22	2	11	71
CM227	NIVO+IPI	>=1% and <50%	24	2	5	60
CM227	NIVO+IPI	>=1% and <50%	26	2	4	55

CM227	NIVO+IPI	>=1% and <50%	28	2	1	51
CM227	NIVO+IPI	>=1% and <50%	30	2	5	50
CM227	NIVO+IPI	>=1% and <50%	32	2	3	44
CM227	NIVO+IPI	>=1% and <50%	34	2	2	41
CM227	NIVO+IPI	>=1% and <50%	36	2	3	39
CM227	NIVO+IPI	>=1% and <50%	38	2	1	36
CM227	NIVO+IPI	>=1% and <50%	40	2	2	35
CM227	NIVO+IPI	>=1% and <50%	42	2	0	33
CM227	NIVO+IPI	>=1% and <50%	44	2	0	33
CM227	NIVO+IPI	>=1% and <50%	46	2	0	33
CM227	NIVO+IPI	>=1% and <50%	48	2	2	33
CM227	NIVO+IPI	>=1% and <50%	50	2	1	31
CM227	NIVO+IPI	>=1% and <50%	52	2	1	30
CM227	NIVO+IPI	>=1% and <50%	54	2	1	29
CM227	NIVO+IPI	>=1% and <50%	56	2	0	28
CM227	NIVO+IPI	>=1% and <50%	58	2	0	28
CM227	NIVO+IPI	>=1% and <50%	60	2	0	28
CM227	NIVO+IPI	>=1% and <50%	62	2	3	27
CM227	NIVO+IPI	>=1% and <50%	64	2	2	24
CM227	NIVO+IPI	>=1% and <50%	66	2	0	21
CM227	NIVO+IPI	>=1% and <50%	68	2	0	16
CM227	NIVO+IPI	>=1% and <50%	70	2	0	12
CM227	NIVO+IPI	>=1% and <50%	72	2	0	7
CM227	NIVO+IPI	>=1% and <50%	74	2	0	1
CM227	NIVO+IPI	all	2	2	0	583
CM227	NIVO+IPI	all	4	2	57	573
CM227	NIVO+IPI	all	6	2	31	506
CM227	NIVO+IPI	all	8	2	43	438
CM227	NIVO+IPI	all	10	2	32	395
CM227	NIVO+IPI	all	12	2	30	363
CM227	NIVO+IPI	all	14	2	20	333
CM227	NIVO+IPI	all	16	2	20	313
CM227	NIVO+IPI	all	18	2	22	293
CM227	NIVO+IPI	all	20	2	19	271
CM227	NIVO+IPI	all	22	2	17	252
CM227	NIVO+IPI	all	24	2	19	235
CM227	NIVO+IPI	all	26	2	9	216
CM227	NIVO+IPI	all	28	2	4	207
CM227	NIVO+IPI	all	30	2	8	203
CM227	NIVO+IPI	all	32	2	12	195
CM227	NIVO+IPI	all	34	2	3	183
CM227	NIVO+IPI	all	36	2	7	180
CM227	NIVO+IPI	all	38	2	4	173
CM227	NIVO+IPI	all	40	2	6	169

CM227	NIVO+IPI	all	42	2	0	163
CM227	NIVO+IPI	all	44	2	5	163
CM227	NIVO+IPI	all	46	2	7	158
CM227	NIVO+IPI	all	48	2	5	151
CM227	NIVO+IPI	all	50	2	8	145
CM227	NIVO+IPI	all	52	2	4	137
CM227	NIVO+IPI	all	54	2	2	133
CM227	NIVO+IPI	all	56	2	3	131
CM227	NIVO+IPI	all	58	2	3	128
CM227	NIVO+IPI	all	60	2	4	125
CM227	NIVO+IPI	all	62	2	6	121
CM227	NIVO+IPI	all	64	2	2	109
CM227	NIVO+IPI	all	66	2	1	101
CM227	NIVO+IPI	all	68	2	1	62
CM227	NIVO+IPI	all	70	2	0	44
CM227	NIVO+IPI	all	72	2	1	28
CM227	NIVO+IPI	all	74	2	0	6
CM227	NIVO+IPI	all	76	2	0	6
CM227	NIVO	>=50%	2	2	0	214
CM227	NIVO	>=50%	4	2	28	212
CM227	NIVO	>=50%	6	2	5	181
CM227	NIVO	>=50%	8	2	9	169
CM227	NIVO	>=50%	10	2	12	160
CM227	NIVO	>=50%	12	2	12	148
CM227	NIVO	>=50%	14	2	13	127
CM227	NIVO	>=50%	16	2	7	114
CM227	NIVO	>=50%	18	2	8	107
CM227	NIVO	>=50%	20	2	5	99
CM227	NIVO	>=50%	22	2	7	94
CM227	NIVO	>=50%	24	2	7	87
CM227	NIVO	>=50%	26	2	3	80
CM227	NIVO	>=50%	28	2	3	77
CM227	NIVO	>=50%	30	2	0	74
CM227	NIVO	>=50%	32	2	4	74
CM227	NIVO	>=50%	34	2	2	70
CM227	NIVO	>=50%	36	2	2	68
CM227	NIVO	>=50%	38	2	1	66
CM227	NIVO	>=50%	40	2	2	65
CM227	NIVO	>=50%	42	2	4	63
CM227	NIVO	>=50%	44	2	2	58
CM227	NIVO	>=50%	46	2	6	56
CM227	NIVO	>=50%	48	2	2	50
CM227	NIVO	>=50%	50	2	0	48
CM227	NIVO	>=50%	52	2	3	48

CM227	NIVO	>=50%	54	2	1	45
CM227	NIVO	>=50%	56	2	1	44
CM227	NIVO	>=50%	58	2	0	43
CM227	NIVO	>=50%	60	2	2	43
CM227	NIVO	>=50%	62	2	2	41
CM227	NIVO	>=50%	64	2	0	36
CM227	NIVO	>=50%	66	2	0	33
CM227	NIVO	>=50%	68	2	0	17
CM227	NIVO	>=50%	70	2	0	12
CM227	NIVO	>=50%	72	2	0	7
CM227	NIVO	>=50%	74	2	0	3
CM227	NIVO	>=50%	76	2	0	3
CM227	NIVO+IPI	>=50%	2	2	0	205
CM227	NIVO+IPI	>=50%	4	2	28	202
CM227	NIVO+IPI	>=50%	6	2	12	172
CM227	NIVO+IPI	>=50%	8	2	8	157
CM227	NIVO+IPI	>=50%	10	2	9	149
CM227	NIVO+IPI	>=50%	12	2	5	140
CM227	NIVO+IPI	>=50%	14	2	5	135
CM227	NIVO+IPI	>=50%	16	2	10	130
CM227	NIVO+IPI	>=50%	18	2	12	120
CM227	NIVO+IPI	>=50%	20	2	4	105
CM227	NIVO+IPI	>=50%	22	2	0	101
CM227	NIVO+IPI	>=50%	24	2	9	101
CM227	NIVO+IPI	>=50%	26	2	1	88
CM227	NIVO+IPI	>=50%	28	2	3	87
CM227	NIVO+IPI	>=50%	30	2	0	84
CM227	NIVO+IPI	>=50%	32	2	5	84
CM227	NIVO+IPI	>=50%	34	2	1	79
CM227	NIVO+IPI	>=50%	36	2	1	78
CM227	NIVO+IPI	>=50%	38	2	1	77
CM227	NIVO+IPI	>=50%	40	2	1	76
CM227	NIVO+IPI	>=50%	42	2	0	75
CM227	NIVO+IPI	>=50%	44	2	1	75
CM227	NIVO+IPI	>=50%	46	2	5	74
CM227	NIVO+IPI	>=50%	48	2	0	69
CM227	NIVO+IPI	>=50%	50	2	2	69
CM227	NIVO+IPI	>=50%	52	2	2	67
CM227	NIVO+IPI	>=50%	54	2	0	65
CM227	NIVO+IPI	>=50%	56	2	2	65
CM227	NIVO+IPI	>=50%	58	2	1	63
CM227	NIVO+IPI	>=50%	60	2	2	62
CM227	NIVO+IPI	>=50%	62	2	2	60
CM227	NIVO+IPI	>=50%	64	2	0	54

CM227	NIVO+IPI	>=50%	66	2	0	50
CM227	NIVO+IPI	>=50%	68	2	0	31
CM227	NIVO+IPI	>=50%	70	2	1	22
CM227	NIVO+IPI	>=50%	72	2	0	13
CM227	NIVO+IPI	>=50%	74	2	0	2
CM227	NIVO	>=1%	2	2	0	396
CM227	NIVO	>=1%	4	2	41	383
CM227	NIVO	>=1%	6	2	34	330
CM227	NIVO	>=1%	8	2	14	281
CM227	NIVO	>=1%	10	2	21	267
CM227	NIVO	>=1%	12	2	20	246
CM227	NIVO	>=1%	14	2	25	220
CM227	NIVO	>=1%	16	2	14	195
CM227	NIVO	>=1%	18	2	15	181
CM227	NIVO	>=1%	20	2	13	166
CM227	NIVO	>=1%	22	2	12	153
CM227	NIVO	>=1%	24	2	12	141
CM227	NIVO	>=1%	26	2	8	129
CM227	NIVO	>=1%	28	2	2	121
CM227	NIVO	>=1%	30	2	9	119
CM227	NIVO	>=1%	32	2	3	110
CM227	NIVO	>=1%	34	2	3	107
CM227	NIVO	>=1%	36	2	5	104
CM227	NIVO	>=1%	38	2	2	99
CM227	NIVO	>=1%	40	2	6	97
CM227	NIVO	>=1%	42	2	4	91
CM227	NIVO	>=1%	44	2	2	87
CM227	NIVO	>=1%	46	2	9	85
CM227	NIVO	>=1%	48	2	4	76
CM227	NIVO	>=1%	50	2	2	72
CM227	NIVO	>=1%	52	2	3	70
CM227	NIVO	>=1%	54	2	2	67
CM227	NIVO	>=1%	56	2	3	65
CM227	NIVO	>=1%	58	2	2	62
CM227	NIVO	>=1%	60	2	3	60
CM227	NIVO	>=1%	62	2	1	57
CM227	NIVO	>=1%	64	2	2	51
CM227	NIVO	>=1%	66	2	0	46
CM227	NIVO	>=1%	68	2	1	27
CM227	NIVO	>=1%	70	2	0	19
CM227	NIVO	>=1%	72	2	2	12
CM227	NIVO	>=1%	74	2	0	3
CM227	NIVO	>=1%	76	2	0	3
CM227	NIVO+IPI	>=1%	2	2	0	396

CM227	NIVO+IPI	>=1%	4	2	48	392
CM227	NIVO+IPI	>=1%	6	2	18	341
CM227	NIVO+IPI	>=1%	8	2	31	296
CM227	NIVO+IPI	>=1%	10	2	16	265
CM227	NIVO+IPI	>=1%	12	2	18	249
CM227	NIVO+IPI	>=1%	14	2	12	231
CM227	NIVO+IPI	>=1%	16	2	14	219
CM227	NIVO+IPI	>=1%	18	2	20	205
CM227	NIVO+IPI	>=1%	20	2	13	185
CM227	NIVO+IPI	>=1%	22	2	15	172
CM227	NIVO+IPI	>=1%	24	2	12	157
CM227	NIVO+IPI	>=1%	26	2	4	145
CM227	NIVO+IPI	>=1%	28	2	3	141
CM227	NIVO+IPI	>=1%	30	2	5	138
CM227	NIVO+IPI	>=1%	32	2	8	133
CM227	NIVO+IPI	>=1%	34	2	4	125
CM227	NIVO+IPI	>=1%	36	2	4	121
CM227	NIVO+IPI	>=1%	38	2	2	117
CM227	NIVO+IPI	>=1%	40	2	2	115
CM227	NIVO+IPI	>=1%	42	2	2	113
CM227	NIVO+IPI	>=1%	44	2	2	111
CM227	NIVO+IPI	>=1%	46	2	4	109
CM227	NIVO+IPI	>=1%	48	2	0	105
CM227	NIVO+IPI	>=1%	50	2	5	104
CM227	NIVO+IPI	>=1%	52	2	2	99
CM227	NIVO+IPI	>=1%	54	2	2	97
CM227	NIVO+IPI	>=1%	56	2	1	95
CM227	NIVO+IPI	>=1%	58	2	2	94
CM227	NIVO+IPI	>=1%	60	2	2	92
CM227	NIVO+IPI	>=1%	62	2	5	89
CM227	NIVO+IPI	>=1%	64	2	0	79
CM227	NIVO+IPI	>=1%	66	2	1	74
CM227	NIVO+IPI	>=1%	68	2	0	47
CM227	NIVO+IPI	>=1%	70	2	0	34
CM227	NIVO+IPI	>=1%	72	2	0	20
CM227	NIVO+IPI	>=1%	74	2	0	3
CM227	NIVO+IPI	>=1%	76	2	0	3
CM227	NIVO+chemo	<1%	2	2	0	177
CM227	NIVO+chemo	<1%	4	2	9	173
CM227	NIVO+chemo	<1%	6	2	14	159
CM227	NIVO+chemo	<1%	8	2	11	139
CM227	NIVO+chemo	<1%	10	2	9	128
CM227	NIVO+chemo	<1%	12	2	16	119
CM227	NIVO+chemo	<1%	14	2	8	102

CM227	NIVO+chemo	<1%	16	2	11	94
CM227	NIVO+chemo	<1%	18	2	7	83
CM227	NIVO+chemo	<1%	20	2	5	76
CM227	NIVO+chemo	<1%	22	2	6	71
CM227	NIVO+chemo	<1%	24	2	6	65
CM227	NIVO+chemo	<1%	26	2	4	58
CM227	NIVO+chemo	<1%	28	2	6	54
CM227	NIVO+chemo	<1%	30	2	6	48
CM227	NIVO+chemo	<1%	32	2	5	42
CM227	NIVO+chemo	<1%	34	2	1	37
CM227	NIVO+chemo	<1%	36	2	1	36
CM227	NIVO+chemo	<1%	38	2	5	34
CM227	NIVO+chemo	<1%	40	2	2	29
CM227	NIVO+chemo	<1%	42	2	1	26
CM227	NIVO+chemo	<1%	44	2	2	25
CM227	NIVO+chemo	<1%	46	2	2	23
CM227	NIVO+chemo	<1%	48	2	1	21
CM227	NIVO+chemo	<1%	50	2	1	20
CM227	NIVO+chemo	<1%	52	2	2	19
CM227	NIVO+chemo	<1%	54	2	1	17
CM227	NIVO+chemo	<1%	56	2	0	16
CM227	NIVO+chemo	<1%	58	2	0	16
CM227	NIVO+chemo	<1%	60	2	0	16
CM227	NIVO+chemo	<1%	62	2	1	16
CM227	NIVO+chemo	<1%	64	2	1	15
CM227	NIVO+chemo	<1%	66	2	0	13
CM227	NIVO+chemo	<1%	68	2	0	7
CM227	NIVO+chemo	<1%	70	2	0	4
CM227	NIVO+chemo	<1%	72	2	0	2
CM227	NIVO+IPI	<1%	2	2	0	187
CM227	NIVO+IPI	<1%	4	2	9	181
CM227	NIVO+IPI	<1%	6	2	12	165
CM227	NIVO+IPI	<1%	8	2	18	142
CM227	NIVO+IPI	<1%	10	2	14	124
CM227	NIVO+IPI	<1%	12	2	8	110
CM227	NIVO+IPI	<1%	14	2	5	102
CM227	NIVO+IPI	<1%	16	2	9	97
CM227	NIVO+IPI	<1%	18	2	3	88
CM227	NIVO+IPI	<1%	20	2	7	85
CM227	NIVO+IPI	<1%	22	2	5	78
CM227	NIVO+IPI	<1%	24	2	2	73
CM227	NIVO+IPI	<1%	26	2	5	71
CM227	NIVO+IPI	<1%	28	2	3	66
CM227	NIVO+IPI	<1%	30	2	2	63

CM227	NIVO+IPI	<1%	32	2	1	61
CM227	NIVO+IPI	<1%	34	2	2	60
CM227	NIVO+IPI	<1%	36	2	3	58
CM227	NIVO+IPI	<1%	38	2	4	55
CM227	NIVO+IPI	<1%	40	2	1	51
CM227	NIVO+IPI	<1%	42	2	0	50
CM227	NIVO+IPI	<1%	44	2	4	49
CM227	NIVO+IPI	<1%	46	2	3	45
CM227	NIVO+IPI	<1%	48	2	1	42
CM227	NIVO+IPI	<1%	50	2	4	41
CM227	NIVO+IPI	<1%	52	2	1	37
CM227	NIVO+IPI	<1%	54	2	0	36
CM227	NIVO+IPI	<1%	56	2	1	36
CM227	NIVO+IPI	<1%	58	2	1	35
CM227	NIVO+IPI	<1%	60	2	3	34
CM227	NIVO+IPI	<1%	62	2	1	31
CM227	NIVO+IPI	<1%	64	2	1	29
CM227	NIVO+IPI	<1%	66	2	1	27
CM227	NIVO+IPI	<1%	68	2	0	15
CM227	NIVO+IPI	<1%	70	2	2	12
CM227	NIVO+IPI	<1%	72	2	0	8
CM227	NIVO+IPI	<1%	74	2	0	3
CM227	NIVO+IPI	<1%	76	2	0	3
IM110	ate	>=1%	2	2	14	277
IM110	ate	>=1%	4	2	21	253
IM110	ate	>=1%	6	2	19	228
IM110	ate	>=1%	8	2	14	206
IM110	ate	>=1%	10	2	19	192
IM110	ate	>=1%	12	2	11	170
IM110	ate	>=1%	14	2	17	159
IM110	ate	>=1%	16	2	4	142
IM110	ate	>=1%	18	2	5	138
IM110	ate	>=1%	20	2	7	133
IM110	ate	>=1%	22	2	13	123
IM110	ate	>=1%	24	2	3	110
IM110	ate	>=1%	26	2	9	103
IM110	ate	>=1%	28	2	5	75
IM110	ate	>=1%	30	2	2	54
IM110	ate	>=1%	32	2	4	46
IM110	ate	>=1%	34	2	1	37
IM110	ate	>=1%	36	2	2	28
IM110	ate	>=1%	38	2	0	20
IM110	ate	>=1%	40	2	0	15
IM110	ate	>=1%	42	2	0	13

IM110	ate	>=1%	44	2	0	8
IM110	ate	>=1%	46	2	1	5
IM110	ate	>=1%	48	2	0	3
IM110	ate	>=1%	50	2	0	3
IM110	ate	>=1%	52	2	0	1
IM110	ate	>=50%	2	2	6	107
IM110	ate	>=50%	4	2	12	95
IM110	ate	>=50%	6	2	4	82
IM110	ate	>=50%	8	2	3	78
IM110	ate	>=50%	10	2	4	75
IM110	ate	>=50%	12	2	3	71
IM110	ate	>=50%	14	2	5	68
IM110	ate	>=50%	16	2	3	63
IM110	ate	>=50%	18	2	2	60
IM110	ate	>=50%	20	2	4	58
IM110	ate	>=50%	22	2	5	54
IM110	ate	>=50%	24	2	0	49
IM110	ate	>=50%	26	2	4	43
IM110	ate	>=50%	28	2	3	38
IM110	ate	>=50%	30	2	1	27
IM110	ate	>=50%	32	2	0	25
IM110	ate	>=50%	34	2	0	22
IM110	ate	>=50%	36	2	1	18
IM110	ate	>=50%	38	2	0	16
IM110	ate	>=50%	40	2	0	14
IM110	ate	>=50%	42	2	0	13
IM110	ate	>=50%	44	2	0	8
IM110	ate	>=50%	46	2	1	5
IM110	ate	>=50%	48	2	0	4
IM110	ate	>=50%	50	2	0	3
IM110	ate	>=50%	52	2	0	1
IM130	ate+chemo	all	2	2	8	451
IM130	ate+chemo	all	4	2	32	423
IM130	ate+chemo	all	6	2	32	381
IM130	ate+chemo	all	8	2	27	347
IM130	ate+chemo	all	10	2	23	318
IM130	ate+chemo	all	12	2	22	293
IM130	ate+chemo	all	14	2	26	268
IM130	ate+chemo	all	16	2	12	213
IM130	ate+chemo	all	18	2	11	171
IM130	ate+chemo	all	20	2	12	129
IM130	ate+chemo	all	22	2	6	91
IM130	ate+chemo	all	24	2	4	62
IM130	ate+chemo	all	26	2	2	40

IM130	ate+chemo	all	28	2	0	19
IM130	ate+chemo	all	30	2	2	10
IM130	ate+chemo	all	32	2	0	4
IM132	ate+car/cis+peme	>=1% and <50%	2	2	1	63
IM132	ate+car/cis+peme	>=1% and <50%	4	2	6	58
IM132	ate+car/cis+peme	>=1% and <50%	6	2	4	51
IM132	ate+car/cis+peme	>=1% and <50%	8	2	6	47
IM132	ate+car/cis+peme	>=1% and <50%	10	2	6	39
IM132	ate+car/cis+peme	>=1% and <50%	12	2	1	32
IM132	ate+car/cis+peme	>=1% and <50%	14	2	7	31
IM132	ate+car/cis+peme	>=1% and <50%	16	2	1	24
IM132	ate+car/cis+peme	>=1% and <50%	18	2	1	23
IM132	ate+car/cis+peme	>=1% and <50%	20	2	3	22
IM132	ate+car/cis+peme	>=1% and <50%	22	2	2	19
IM132	ate+car/cis+peme	>=1% and <50%	24	2	1	17
IM132	ate+car/cis+peme	>=1% and <50%	26	2	3	16
IM132	ate+car/cis+peme	>=1% and <50%	28	2	0	12
IM132	ate+car/cis+peme	>=1% and <50%	30	2	1	11
IM132	ate+car/cis+peme	>=1% and <50%	32	2	0	5
IM132	ate+car/cis+peme	>=1% and <50%	34	2	0	5
IM132	ate+car/cis+peme	>=1% and <50%	36	2	0	1
IM132	ate+car/cis+peme	all	2	2	8	292
IM132	ate+car/cis+peme	all	4	2	19	273
IM132	ate+car/cis+peme	all	6	2	19	242
IM132	ate+car/cis+peme	all	8	2	24	223
IM132	ate+car/cis+peme	all	10	2	14	195
IM132	ate+car/cis+peme	all	12	2	16	181
IM132	ate+car/cis+peme	all	14	2	16	165
IM132	ate+car/cis+peme	all	16	2	9	149
IM132	ate+car/cis+peme	all	18	2	7	140
IM132	ate+car/cis+peme	all	20	2	16	133
IM132	ate+car/cis+peme	all	22	2	5	115
IM132	ate+car/cis+peme	all	24	2	9	110
IM132	ate+car/cis+peme	all	26	2	6	101
IM132	ate+car/cis+peme	all	28	2	5	95
IM132	ate+car/cis+peme	all	30	2	3	62
IM132	ate+car/cis+peme	all	32	2	2	36
IM132	ate+car/cis+peme	all	34	2	0	23
IM132	ate+car/cis+peme	all	36	2	0	7
IM132	ate+car/cis+peme	>=50%	2	2	0	25
IM132	ate+car/cis+peme	>=50%	4	2	1	24
IM132	ate+car/cis+peme	>=50%	6	2	0	22
IM132	ate+car/cis+peme	>=50%	8	2	1	22
IM132	ate+car/cis+peme	>=50%	10	2	0	21

IM132	ate+car/cis+peme	>=50%	12	2	1	21
IM132	ate+car/cis+peme	>=50%	14	2	1	20
IM132	ate+car/cis+peme	>=50%	16	2	1	19
IM132	ate+car/cis+peme	>=50%	18	2	0	18
IM132	ate+car/cis+peme	>=50%	20	2	1	18
IM132	ate+car/cis+peme	>=50%	22	2	0	17
IM132	ate+car/cis+peme	>=50%	24	2	2	17
IM132	ate+car/cis+peme	>=50%	26	2	0	15
IM132	ate+car/cis+peme	>=50%	28	2	1	15
IM132	ate+car/cis+peme	>=50%	30	2	1	10
IM132	ate+car/cis+peme	>=50%	32	2	0	5
IM132	ate+car/cis+peme	>=50%	34	2	0	3
IM132	ate+car/cis+peme	<1%	2	2	2	88
IM132	ate+car/cis+peme	<1%	4	2	8	82
IM132	ate+car/cis+peme	<1%	6	2	5	70
IM132	ate+car/cis+peme	<1%	8	2	10	65
IM132	ate+car/cis+peme	<1%	10	2	1	53
IM132	ate+car/cis+peme	<1%	12	2	5	52
IM132	ate+car/cis+peme	<1%	14	2	6	47
IM132	ate+car/cis+peme	<1%	16	2	1	41
IM132	ate+car/cis+peme	<1%	18	2	2	40
IM132	ate+car/cis+peme	<1%	20	2	3	38
IM132	ate+car/cis+peme	<1%	22	2	1	35
IM132	ate+car/cis+peme	<1%	24	2	3	34
IM132	ate+car/cis+peme	<1%	26	2	2	31
IM132	ate+car/cis+peme	<1%	28	2	0	29
IM132	ate+car/cis+peme	<1%	30	2	0	20
IM132	ate+car/cis+peme	<1%	32	2	1	12
IM132	ate+car/cis+peme	<1%	34	2	0	10
IM132	ate+car/cis+peme	<1%	36	2	1	6
IM150	ate+beva+car+pac	>=1%	2	2	10	192
IM150	ate+beva+car+pac	>=1%	4	2	8	179
IM150	ate+beva+car+pac	>=1%	6	2	2	168
IM150	ate+beva+car+pac	>=1%	8	2	15	162
IM150	ate+beva+car+pac	>=1%	10	2	8	147
IM150	ate+beva+car+pac	>=1%	12	2	10	139
IM150	ate+beva+car+pac	>=1%	14	2	7	129
IM150	ate+beva+car+pac	>=1%	16	2	6	122
IM150	ate+beva+car+pac	>=1%	18	2	8	116
IM150	ate+beva+car+pac	>=1%	20	2	6	108
IM150	ate+beva+car+pac	>=1%	22	2	6	102
IM150	ate+beva+car+pac	>=1%	24	2	6	96
IM150	ate+beva+car+pac	>=1%	26	2	1	90
IM150	ate+beva+car+pac	>=1%	28	2	10	88

IM150	ate+beva+car+pac	>=1%	30	2	1	77
IM150	ate+beva+car+pac	>=1%	32	2	4	75
IM150	ate+beva+car+pac	>=1%	34	2	5	65
IM150	ate+beva+car+pac	>=1%	36	2	2	54
IM150	ate+beva+car+pac	>=1%	38	2	1	49
IM150	ate+beva+car+pac	>=1%	40	2	1	40
IM150	ate+beva+car+pac	>=1%	42	2	1	30
IM150	ate+beva+car+pac	>=1%	44	2	1	20
IM150	ate+beva+car+pac	>=1%	46	2	0	11
IM150	ate+beva+car+pac	>=1%	48	2	0	6
IM150	ate+beva+car+pac	>=1%	50	2	0	1
IM150	ate+car+pac	>=1%	2	2	6	185
IM150	ate+car+pac	>=1%	4	2	5	175
IM150	ate+car+pac	>=1%	6	2	11	169
IM150	ate+car+pac	>=1%	8	2	13	157
IM150	ate+car+pac	>=1%	10	2	3	143
IM150	ate+car+pac	>=1%	12	2	11	139
IM150	ate+car+pac	>=1%	14	2	8	128
IM150	ate+car+pac	>=1%	16	2	6	120
IM150	ate+car+pac	>=1%	18	2	5	114
IM150	ate+car+pac	>=1%	20	2	5	109
IM150	ate+car+pac	>=1%	22	2	8	104
IM150	ate+car+pac	>=1%	24	2	4	96
IM150	ate+car+pac	>=1%	26	2	9	92
IM150	ate+car+pac	>=1%	28	2	6	82
IM150	ate+car+pac	>=1%	30	2	5	76
IM150	ate+car+pac	>=1%	32	2	3	71
IM150	ate+car+pac	>=1%	34	2	2	64
IM150	ate+car+pac	>=1%	36	2	0	54
IM150	ate+car+pac	>=1%	38	2	0	45
IM150	ate+car+pac	>=1%	40	2	1	36
IM150	ate+car+pac	>=1%	42	2	2	25
IM150	ate+car+pac	>=1%	44	2	0	13
IM150	ate+car+pac	>=1%	46	2	1	7
IM150	ate+car+pac	>=1%	48	2	0	2
IM150	ate+car+pac	>=1%	50	2	0	1
IM150	ate+beva+car+pac	all	2	2	13	359
IM150	ate+beva+car+pac	all	4	2	15	339
IM150	ate+beva+car+pac	all	6	2	14	317
IM150	ate+beva+car+pac	all	8	2	22	298
IM150	ate+beva+car+pac	all	10	2	21	276
IM150	ate+beva+car+pac	all	12	2	15	254
IM150	ate+beva+car+pac	all	14	2	23	236
IM150	ate+beva+car+pac	all	16	2	15	212

IM150	ate+beva+car+pac	all	18	2	13	197
IM150	ate+beva+car+pac	all	20	2	17	184
IM150	ate+beva+car+pac	all	22	2	12	167
IM150	ate+beva+car+pac	all	24	2	11	155
IM150	ate+beva+car+pac	all	26	2	12	144
IM150	ate+beva+car+pac	all	28	2	13	132
IM150	ate+beva+car+pac	all	30	2	7	119
IM150	ate+beva+car+pac	all	32	2	6	111
IM150	ate+beva+car+pac	all	34	2	6	98
IM150	ate+beva+car+pac	all	36	2	3	84
IM150	ate+beva+car+pac	all	38	2	2	71
IM150	ate+beva+car+pac	all	40	2	4	58
IM150	ate+beva+car+pac	all	42	2	0	41
IM150	ate+beva+car+pac	all	44	2	2	27
IM150	ate+beva+car+pac	all	46	2	0	15
IM150	ate+beva+car+pac	all	48	2	0	7
IM150	ate+beva+car+pac	all	50	2	0	1
IM150	ate+car+pac	all	2	2	7	350
IM150	ate+car+pac	all	4	2	20	335
IM150	ate+car+pac	all	6	2	22	311
IM150	ate+car+pac	all	8	2	28	286
IM150	ate+car+pac	all	10	2	21	256
IM150	ate+car+pac	all	12	2	18	234
IM150	ate+car+pac	all	14	2	12	216
IM150	ate+car+pac	all	16	2	17	203
IM150	ate+car+pac	all	18	2	16	186
IM150	ate+car+pac	all	20	2	6	169
IM150	ate+car+pac	all	22	2	15	163
IM150	ate+car+pac	all	24	2	11	148
IM150	ate+car+pac	all	26	2	11	137
IM150	ate+car+pac	all	28	2	9	126
IM150	ate+car+pac	all	30	2	9	117
IM150	ate+car+pac	all	32	2	7	108
IM150	ate+car+pac	all	34	2	6	97
IM150	ate+car+pac	all	36	2	1	81
IM150	ate+car+pac	all	38	2	0	64
IM150	ate+car+pac	all	40	2	1	53
IM150	ate+car+pac	all	42	2	2	37
IM150	ate+car+pac	all	44	2	0	20
IM150	ate+car+pac	all	46	2	2	12
IM150	ate+car+pac	all	48	2	0	5
IM150	ate+car+pac	all	50	2	0	2
IM150	ate+beva+car+pac	>=50%	2	2	4	71
IM150	ate+beva+car+pac	>=50%	4	2	3	67

IM150	ate+beva+car+pac	>=50%	6	2	0	64
IM150	ate+beva+car+pac	>=50%	8	2	7	63
IM150	ate+beva+car+pac	>=50%	10	2	2	55
IM150	ate+beva+car+pac	>=50%	12	2	1	53
IM150	ate+beva+car+pac	>=50%	14	2	2	52
IM150	ate+beva+car+pac	>=50%	16	2	3	50
IM150	ate+beva+car+pac	>=50%	18	2	1	47
IM150	ate+beva+car+pac	>=50%	20	2	2	46
IM150	ate+beva+car+pac	>=50%	22	2	1	44
IM150	ate+beva+car+pac	>=50%	24	2	2	43
IM150	ate+beva+car+pac	>=50%	26	2	1	41
IM150	ate+beva+car+pac	>=50%	28	2	4	40
IM150	ate+beva+car+pac	>=50%	30	2	0	36
IM150	ate+beva+car+pac	>=50%	32	2	2	36
IM150	ate+beva+car+pac	>=50%	34	2	1	34
IM150	ate+beva+car+pac	>=50%	36	2	1	29
IM150	ate+beva+car+pac	>=50%	38	2	0	24
IM150	ate+beva+car+pac	>=50%	40	2	1	20
IM150	ate+beva+car+pac	>=50%	42	2	1	13
IM150	ate+beva+car+pac	>=50%	44	2	1	10
IM150	ate+beva+car+pac	>=50%	46	2	0	8
IM150	ate+beva+car+pac	>=50%	48	2	0	5
IM150	ate+beva+car+pac	>=50%	50	2	0	1
IM150	ate+beva+car+pac	>=50%	52	2	0	0
IM150	ate+car+pac	>=50%	2	2	2	63
IM150	ate+car+pac	>=50%	4	2	3	60
IM150	ate+car+pac	>=50%	6	2	5	56
IM150	ate+car+pac	>=50%	8	2	2	51
IM150	ate+car+pac	>=50%	10	2	0	48
IM150	ate+car+pac	>=50%	12	2	5	48
IM150	ate+car+pac	>=50%	14	2	1	43
IM150	ate+car+pac	>=50%	16	2	1	42
IM150	ate+car+pac	>=50%	18	2	2	41
IM150	ate+car+pac	>=50%	20	2	4	39
IM150	ate+car+pac	>=50%	22	2	3	35
IM150	ate+car+pac	>=50%	24	2	0	32
IM150	ate+car+pac	>=50%	26	2	1	32
IM150	ate+car+pac	>=50%	28	2	2	31
IM150	ate+car+pac	>=50%	30	2	0	29
IM150	ate+car+pac	>=50%	32	2	2	29
IM150	ate+car+pac	>=50%	34	2	1	27
IM150	ate+car+pac	>=50%	36	2	0	21
IM150	ate+car+pac	>=50%	38	2	0	16
IM150	ate+car+pac	>=50%	40	2	1	13

IM150	ate+car+pac	>=50%	42	2	1	8
IM150	ate+car+pac	>=50%	44	2	0	4
IM150	ate+car+pac	>=50%	46	2	0	3
IM150	ate+car+pac	>=50%	48	2	0	2
IM150	ate+car+pac	>=50%	50	2	0	0
IM150	ate+beva+car+pac	<1%	2	2	6	167
IM150	ate+beva+car+pac	<1%	4	2	6	159
IM150	ate+beva+car+pac	<1%	6	2	9	150
IM150	ate+beva+car+pac	<1%	8	2	15	136
IM150	ate+beva+car+pac	<1%	10	2	8	120
IM150	ate+beva+car+pac	<1%	12	2	8	111
IM150	ate+beva+car+pac	<1%	14	2	10	103
IM150	ate+beva+car+pac	<1%	16	2	9	93
IM150	ate+beva+car+pac	<1%	18	2	9	84
IM150	ate+beva+car+pac	<1%	20	2	5	75
IM150	ate+beva+car+pac	<1%	22	2	11	70
IM150	ate+beva+car+pac	<1%	24	2	6	59
IM150	ate+beva+car+pac	<1%	26	2	6	53
IM150	ate+beva+car+pac	<1%	28	2	5	47
IM150	ate+beva+car+pac	<1%	30	2	7	42
IM150	ate+beva+car+pac	<1%	32	2	2	35
IM150	ate+beva+car+pac	<1%	34	2	0	33
IM150	ate+beva+car+pac	<1%	36	2	1	29
IM150	ate+beva+car+pac	<1%	38	2	2	22
IM150	ate+beva+car+pac	<1%	40	2	1	17
IM150	ate+beva+car+pac	<1%	42	2	0	12
IM150	ate+beva+car+pac	<1%	44	2	0	7
IM150	ate+beva+car+pac	<1%	46	2	0	3
IM150	ate+beva+car+pac	<1%	48	2	0	1
IM150	ate+car+pac	<1%	2	2	4	164
IM150	ate+car+pac	<1%	4	2	11	156
IM150	ate+car+pac	<1%	6	2	12	142
IM150	ate+car+pac	<1%	8	2	15	128
IM150	ate+car+pac	<1%	10	2	17	111
IM150	ate+car+pac	<1%	12	2	5	93
IM150	ate+car+pac	<1%	14	2	4	88
IM150	ate+car+pac	<1%	16	2	9	84
IM150	ate+car+pac	<1%	18	2	13	75
IM150	ate+car+pac	<1%	20	2	3	62
IM150	ate+car+pac	<1%	22	2	6	59
IM150	ate+car+pac	<1%	24	2	5	53
IM150	ate+car+pac	<1%	26	2	6	48
IM150	ate+car+pac	<1%	28	2	2	42
IM150	ate+car+pac	<1%	30	2	4	40

IM150	ate+car+pac	<1%	32	2	4	36
IM150	ate+car+pac	<1%	34	2	0	30
IM150	ate+car+pac	<1%	36	2	1	27
IM150	ate+car+pac	<1%	38	2	1	19
IM150	ate+car+pac	<1%	40	2	0	16
IM150	ate+car+pac	<1%	42	2	0	11
IM150	ate+car+pac	<1%	44	2	0	7
IM150	ate+car+pac	<1%	46	2	1	5
IM150	ate+car+pac	<1%	48	2	0	2
IM150	ate+car+pac	<1%	50	2	0	1
IM150	ate+car+pac	<1%	52	2	0	0
KN042	pem	>=1%	2	2	0	637
KN042	pem	>=1%	4	2	66	622
KN042	pem	>=1%	6	2	63	540
KN042	pem	>=1%	8	2	40	463
KN042	pem	>=1%	10	2	35	423
KN042	pem	>=1%	12	2	33	388
KN042	pem	>=1%	14	2	20	355
KN042	pem	>=1%	16	2	22	335
KN042	pem	>=1%	18	2	20	313
KN042	pem	>=1%	20	2	13	293
KN042	pem	>=1%	22	2	16	280
KN042	pem	>=1%	24	2	19	264
KN042	pem	>=1%	26	2	20	245
KN042	pem	>=1%	28	2	16	224
KN042	pem	>=1%	30	2	20	207
KN042	pem	>=1%	32	2	9	186
KN042	pem	>=1%	34	2	16	177
KN042	pem	>=1%	36	2	10	161
KN042	pem	>=1%	38	2	7	151
KN042	pem	>=1%	40	2	9	144
KN042	pem	>=1%	42	2	7	135
KN042	pem	>=1%	44	2	6	128
KN042	pem	>=1%	46	2	3	122
KN042	pem	>=1%	48	2	0	119
KN042	pem	>=1%	50	2	3	119
KN042	pem	>=1%	52	2	4	110
KN042	pem	>=1%	54	2	2	101
KN042	pem	>=1%	56	2	3	93
KN042	pem	>=1%	58	2	5	83
KN042	pem	>=1%	60	2	2	69
KN042	pem	>=1%	62	2	2	59
KN042	pem	>=1%	64	2	2	49
KN042	pem	>=1%	66	2	0	39

KN042	pem	>=1%	68	2	0	28
KN042	pem	>=1%	70	2	0	19
KN042	pem	>=1%	72	2	0	14
KN042	pem	>=1%	74	2	0	3
KN042	pem	>=1%	76	2	0	1
KN042	pem	>=50%	2	2	0	299
KN042	pem	>=50%	4	2	30	291
KN042	pem	>=50%	6	2	23	254
KN042	pem	>=50%	8	2	17	224
KN042	pem	>=50%	10	2	13	207
KN042	pem	>=50%	12	2	9	194
KN042	pem	>=50%	14	2	10	185
KN042	pem	>=50%	16	2	12	175
KN042	pem	>=50%	18	2	11	163
KN042	pem	>=50%	20	2	7	152
KN042	pem	>=50%	22	2	9	145
KN042	pem	>=50%	24	2	5	136
KN042	pem	>=50%	26	2	9	131
KN042	pem	>=50%	28	2	8	122
KN042	pem	>=50%	30	2	5	114
KN042	pem	>=50%	32	2	8	109
KN042	pem	>=50%	34	2	5	101
KN042	pem	>=50%	36	2	5	95
KN042	pem	>=50%	38	2	4	90
KN042	pem	>=50%	40	2	3	86
KN042	pem	>=50%	42	2	6	83
KN042	pem	>=50%	44	2	4	77
KN042	pem	>=50%	46	2	1	73
KN042	pem	>=50%	48	2	0	72
KN042	pem	>=50%	50	2	2	72
KN042	pem	>=50%	52	2	3	66
KN042	pem	>=50%	54	2	0	59
KN042	pem	>=50%	56	2	1	56
KN042	pem	>=50%	58	2	2	51
KN042	pem	>=50%	60	2	2	44
KN042	pem	>=50%	62	2	1	37
KN042	pem	>=50%	64	2	2	30
KN042	pem	>=50%	66	2	0	21
KN042	pem	>=50%	68	2	0	16
KN042	pem	>=50%	70	2	0	11
KN042	pem	>=50%	72	2	0	6
KN042	pem	>=50%	74	2	0	2
KN042	pem	>=50%	76	2	0	1
KN042	pem	>=1% and <50%	2	2	0	338

KN042	pem	>=1% and <50%	4	2	41	329
KN042	pem	>=1% and <50%	6	2	32	279
KN042	pem	>=1% and <50%	8	2	23	239
KN042	pem	>=1% and <50%	10	2	24	216
KN042	pem	>=1% and <50%	12	2	15	190
KN042	pem	>=1% and <50%	14	2	14	175
KN042	pem	>=1% and <50%	16	2	13	161
KN042	pem	>=1% and <50%	18	2	10	148
KN042	pem	>=1% and <50%	20	2	7	138
KN042	pem	>=1% and <50%	22	2	8	130
KN042	pem	>=1% and <50%	24	2	6	121
KN042	pem	>=1% and <50%	26	2	13	114
KN042	pem	>=1% and <50%	28	2	13	101
KN042	pem	>=1% and <50%	30	2	8	87
KN042	pem	>=1% and <50%	32	2	5	79
KN042	pem	>=1% and <50%	34	2	11	74
KN042	pem	>=1% and <50%	36	2	3	63
KN042	pem	>=1% and <50%	38	2	1	60
KN042	pem	>=1% and <50%	40	2	8	59
KN042	pem	>=1% and <50%	42	2	2	51
KN042	pem	>=1% and <50%	44	2	2	49
KN042	pem	>=1% and <50%	46	2	0	47
KN042	pem	>=1% and <50%	48	2	0	47
KN042	pem	>=1% and <50%	50	2	0	47
KN042	pem	>=1% and <50%	52	2	1	46
KN042	pem	>=1% and <50%	54	2	4	43
KN042	pem	>=1% and <50%	56	2	2	37
KN042	pem	>=1% and <50%	58	2	2	32
KN042	pem	>=1% and <50%	60	2	2	27
KN042	pem	>=1% and <50%	62	2	0	22
KN042	pem	>=1% and <50%	64	2	0	18
KN042	pem	>=1% and <50%	66	2	0	15
KN042	pem	>=1% and <50%	68	2	0	12
KN042	pem	>=1% and <50%	70	2	0	8
KN042	pem	>=1% and <50%	72	2	0	4
KN042	pem	>=1% and <50%	74	2	0	1
KN189	pem+chemo	>=1% and <50%	2	2	0	128
KN189	pem+chemo	>=1% and <50%	4	2	5	127
KN189	pem+chemo	>=1% and <50%	6	2	8	121
KN189	pem+chemo	>=1% and <50%	8	2	6	112
KN189	pem+chemo	>=1% and <50%	10	2	5	105
KN189	pem+chemo	>=1% and <50%	12	2	8	99
KN189	pem+chemo	>=1% and <50%	14	2	4	91
KN189	pem+chemo	>=1% and <50%	16	2	6	86

KN189	pem+chemo	>=1% and <50%	18	2	3	79
KN189	pem+chemo	>=1% and <50%	20	2	6	75
KN189	pem+chemo	>=1% and <50%	22	2	6	68
KN189	pem+chemo	>=1% and <50%	24	2	4	61
KN189	pem+chemo	>=1% and <50%	26	2	6	56
KN189	pem+chemo	>=1% and <50%	28	2	5	50
KN189	pem+chemo	>=1% and <50%	30	2	3	45
KN189	pem+chemo	>=1% and <50%	32	2	2	42
KN189	pem+chemo	>=1% and <50%	34	2	3	40
KN189	pem+chemo	>=1% and <50%	36	2	3	37
KN189	pem+chemo	>=1% and <50%	38	2	1	34
KN189	pem+chemo	>=1% and <50%	40	2	2	33
KN189	pem+chemo	>=1% and <50%	42	2	2	31
KN189	pem+chemo	>=1% and <50%	44	2	2	29
KN189	pem+chemo	>=1% and <50%	46	2	1	27
KN189	pem+chemo	>=1% and <50%	48	2	1	26
KN189	pem+chemo	>=1% and <50%	50	2	0	25
KN189	pem+chemo	>=1% and <50%	52	2	1	25
KN189	pem+chemo	>=1% and <50%	54	2	0	24
KN189	pem+chemo	>=1% and <50%	56	2	2	24
KN189	pem+chemo	>=1% and <50%	58	2	0	22
KN189	pem+chemo	>=1% and <50%	60	2	0	22
KN189	pem+chemo	>=1% and <50%	62	2	0	22
KN189	pem+chemo	>=1% and <50%	64	2	0	22
KN189	pem+chemo	>=1% and <50%	66	2	1	22
KN189	pem+chemo	>=1% and <50%	68	2	0	20
KN189	pem+chemo	>=1% and <50%	70	2	1	20
KN189	pem+chemo	>=1% and <50%	72	2	1	19
KN189	pem+chemo	all	2	2	0	410
KN189	pem+chemo	all	4	2	15	406
KN189	pem+chemo	all	6	2	24	387
KN189	pem+chemo	all	8	2	21	359
KN189	pem+chemo	all	10	2	16	334
KN189	pem+chemo	all	12	2	27	313
KN189	pem+chemo	all	14	2	16	283
KN189	pem+chemo	all	16	2	16	267
KN189	pem+chemo	all	18	2	15	250
KN189	pem+chemo	all	20	2	14	234
KN189	pem+chemo	all	22	2	20	219
KN189	pem+chemo	all	24	2	14	198
KN189	pem+chemo	all	26	2	12	184
KN189	pem+chemo	all	28	2	14	172
KN189	pem+chemo	all	30	2	8	158
KN189	pem+chemo	all	32	2	10	150

KN189	pem+chemo	all	34	2	4	140
KN189	pem+chemo	all	36	2	10	136
KN189	pem+chemo	all	38	2	8	126
KN189	pem+chemo	all	40	2	8	118
KN189	pem+chemo	all	42	2	6	110
KN189	pem+chemo	all	44	2	4	104
KN189	pem+chemo	all	46	2	4	100
KN189	pem+chemo	all	48	2	6	96
KN189	pem+chemo	all	50	2	2	90
KN189	pem+chemo	all	52	2	4	88
KN189	pem+chemo	all	54	2	6	84
KN189	pem+chemo	all	56	2	0	78
KN189	pem+chemo	all	58	2	2	78
KN189	pem+chemo	all	60	2	2	76
KN189	pem+chemo	all	62	2	2	74
KN189	pem+chemo	all	64	2	0	61
KN189	pem+chemo	all	66	2	1	49
KN189	pem+chemo	all	68	2	2	37
KN189	pem+chemo	all	70	2	0	23
KN189	pem+chemo	all	72	2	0	9
KN189	pem+chemo	all	74	2	0	0
KN189	pem+chemo	>=1%	2	2	0	260
KN189	pem+chemo	>=1%	4	2	12	258
KN189	pem+chemo	>=1%	6	2	12	244
KN189	pem+chemo	>=1%	8	2	13	230
KN189	pem+chemo	>=1%	10	2	13	215
KN189	pem+chemo	>=1%	12	2	12	200
KN189	pem+chemo	>=1%	14	2	13	186
KN189	pem+chemo	>=1%	16	2	4	173
KN189	pem+chemo	>=1%	18	2	7	167
KN189	pem+chemo	>=1%	20	2	9	159
KN189	pem+chemo	>=1%	22	2	13	149
KN189	pem+chemo	>=1%	24	2	11	135
KN189	pem+chemo	>=1%	26	2	10	123
KN189	pem+chemo	>=1%	28	2	6	113
KN189	pem+chemo	>=1%	30	2	5	107
KN189	pem+chemo	>=1%	32	2	5	102
KN189	pem+chemo	>=1%	34	2	2	97
KN189	pem+chemo	>=1%	36	2	7	95
KN189	pem+chemo	>=1%	38	2	3	88
KN189	pem+chemo	>=1%	40	2	2	85
KN189	pem+chemo	>=1%	42	2	5	83
KN189	pem+chemo	>=1%	44	2	5	78
KN189	pem+chemo	>=1%	46	2	2	73

KN189	pem+chemo	>=1%	48	2	3	71
KN189	pem+chemo	>=1%	50	2	2	68
KN189	pem+chemo	>=1%	52	2	1	66
KN189	pem+chemo	>=1%	54	2	5	65
KN189	pem+chemo	>=1%	56	2	0	60
KN189	pem+chemo	>=1%	58	2	0	60
KN189	pem+chemo	>=1%	60	2	0	60
KN189	pem+chemo	>=1%	62	2	0	60
KN189	pem+chemo	>=1%	64	2	1	51
KN189	pem+chemo	>=1%	66	2	1	40
KN189	pem+chemo	>=1%	68	2	0	30
KN189	pem+chemo	>=1%	70	2	2	19
KN189	pem+chemo	>=1%	72	2	0	8
KN189	pem+chemo	>=1%	74	2	0	0
KN189	pem+chemo	>=50%	2	2	0	132
KN189	pem+chemo	>=50%	4	2	8	131
KN189	pem+chemo	>=50%	6	2	2	121
KN189	pem+chemo	>=50%	8	2	6	117
KN189	pem+chemo	>=50%	10	2	3	109
KN189	pem+chemo	>=50%	12	2	8	104
KN189	pem+chemo	>=50%	14	2	6	95
KN189	pem+chemo	>=50%	16	2	2	89
KN189	pem+chemo	>=50%	18	2	5	87
KN189	pem+chemo	>=50%	20	2	2	82
KN189	pem+chemo	>=50%	22	2	6	79
KN189	pem+chemo	>=50%	24	2	7	73
KN189	pem+chemo	>=50%	26	2	3	66
KN189	pem+chemo	>=50%	28	2	2	63
KN189	pem+chemo	>=50%	30	2	1	61
KN189	pem+chemo	>=50%	32	2	3	60
KN189	pem+chemo	>=50%	34	2	0	57
KN189	pem+chemo	>=50%	36	2	3	57
KN189	pem+chemo	>=50%	38	2	2	54
KN189	pem+chemo	>=50%	40	2	3	52
KN189	pem+chemo	>=50%	42	2	2	49
KN189	pem+chemo	>=50%	44	2	2	47
KN189	pem+chemo	>=50%	46	2	1	45
KN189	pem+chemo	>=50%	48	2	3	44
KN189	pem+chemo	>=50%	50	2	0	41
KN189	pem+chemo	>=50%	52	2	1	41
KN189	pem+chemo	>=50%	54	2	3	40
KN189	pem+chemo	>=50%	56	2	0	37
KN189	pem+chemo	>=50%	58	2	0	37
KN189	pem+chemo	>=50%	60	2	1	37

KN189	pem+chemo	>=50%	62	2	1	36
KN189	pem+chemo	>=50%	64	2	0	35
KN189	pem+chemo	>=50%	66	2	0	35
KN189	pem+chemo	>=50%	68	2	0	34
KN189	pem+chemo	>=50%	70	2	3	34
KN189	pem+chemo	>=50%	72	2	0	31
KN189	pem+chemo	<1%	2	2	0	127
KN189	pem+chemo	<1%	4	2	6	125
KN189	pem+chemo	<1%	6	2	12	117
KN189	pem+chemo	<1%	8	2	4	104
KN189	pem+chemo	<1%	10	2	8	97
KN189	pem+chemo	<1%	12	2	7	88
KN189	pem+chemo	<1%	14	2	7	79
KN189	pem+chemo	<1%	16	2	6	72
KN189	pem+chemo	<1%	18	2	6	66
KN189	pem+chemo	<1%	20	2	4	60
KN189	pem+chemo	<1%	22	2	6	56
KN189	pem+chemo	<1%	24	2	3	50
KN189	pem+chemo	<1%	26	2	3	47
KN189	pem+chemo	<1%	28	2	4	44
KN189	pem+chemo	<1%	30	2	3	40
KN189	pem+chemo	<1%	32	2	3	37
KN189	pem+chemo	<1%	34	2	1	33
KN189	pem+chemo	<1%	36	2	3	32
KN189	pem+chemo	<1%	38	2	3	29
KN189	pem+chemo	<1%	40	2	3	26
KN189	pem+chemo	<1%	42	2	2	23
KN189	pem+chemo	<1%	44	2	0	21
KN189	pem+chemo	<1%	46	2	1	21
KN189	pem+chemo	<1%	48	2	3	20
KN189	pem+chemo	<1%	50	2	0	17
KN189	pem+chemo	<1%	52	2	0	17
KN189	pem+chemo	<1%	54	2	1	17
KN189	pem+chemo	<1%	56	2	2	16
KN189	pem+chemo	<1%	58	2	1	14
KN189	pem+chemo	<1%	60	2	2	13
KN189	pem+chemo	<1%	62	2	0	11
KN189	pem+chemo	<1%	64	2	1	11
KN189	pem+chemo	<1%	66	2	0	10
KN189	pem+chemo	<1%	68	2	0	9
KN189	pem+chemo	<1%	70	2	0	9
KN189	pem+chemo	<1%	72	2	0	9
KN598	pem	>=50%	2	2	15	284
KN598	pem	>=50%	4	2	19	262

KN598	pem	>=50%	6	2	14	239
KN598	pem	>=50%	8	2	10	223
KN598	pem	>=50%	10	2	10	213
KN598	pem	>=50%	12	2	18	203
KN598	pem	>=50%	14	2	8	185
KN598	pem	>=50%	16	2	9	168
KN598	pem	>=50%	18	2	11	141
KN598	pem	>=50%	20	2	2	111
KN598	pem	>=50%	22	2	8	92
KN598	pem	>=50%	24	2	2	66
KN598	pem	>=50%	26	2	2	41
KN598	pem	>=50%	28	2	1	22
KN598	pem	>=50%	30	2	0	10
KN598	pem	>=50%	32	2	0	2
KN598	pem+ipi	>=50%	2	2	18	284
KN598	pem+ipi	>=50%	4	2	19	258
KN598	pem+ipi	>=50%	6	2	17	235
KN598	pem+ipi	>=50%	8	2	9	218
KN598	pem+ipi	>=50%	10	2	13	209
KN598	pem+ipi	>=50%	12	2	16	196
KN598	pem+ipi	>=50%	14	2	15	180
KN598	pem+ipi	>=50%	16	2	7	153
KN598	pem+ipi	>=50%	18	2	4	130
KN598	pem+ipi	>=50%	20	2	6	100
KN598	pem+ipi	>=50%	22	2	1	72
KN598	pem+ipi	>=50%	24	2	3	55
KN598	pem+ipi	>=50%	26	2	2	37
KN598	pem+ipi	>=50%	28	2	0	17
KN598	pem+ipi	>=50%	30	2	0	5

Table S5 AIC value for PFS fit

Fractional Polynomial	AIC Value
First order FP,p1=0	5313.96
First order FP,p1=1	5055.5
First order FP,p1=-2	811100.17
First order FP,p1=-1	5317.85
First order FP,p1=-0.5	5380.47
First order FP,p1=0.5	5169.34
First order FP,p1=2	5042.41
First order FP,p1=3	5145.17
Second order FP,p1=-2, p2=-2	2743.83
Second order FP,p1=-2, p2=-1	2722.92
Second order FP,p1=-2, p2=-0.5	2770
Second order FP,p1=-2, p2=0	2884.92
Second order FP,p1=-2, p2=0.5	3071.95
Second order FP,p1=-2, p2=1	3307.34
Second order FP,p1=-2, p2=2	589964.32
Second order FP,p1=-2, p2=3	830375.48
Second order FP,p1=-1, p2=-1	2830.38
Second order FP,p1=-1, p2=-0.5	2960.12
Second order FP,p1=-1, p2=0	3154.28
Second order FP,p1=-1, p2=0.5	3402.83
Second order FP,p1=-1, p2=1	3675.86
Second order FP,p1=-1, p2=2	4164.95
Second order FP,p1=-1, p2=3	4498.18
Second order FP,p1=-0.5, p2=-0.5	3133.79
Second order FP,p1=-0.5, p2=0	3363.63
Second order FP,p1=-0.5, p2=0.5	3632.78
Second order FP,p1=-0.5, p2=1	3909.97
Second order FP,p1=-0.5, p2=2	4377.57
Second order FP,p1=-0.5, p2=3	4681.74
Second order FP,p1=0, p2=0	3616.18
Second order FP,p1=0, p2=0.5	3889.11
Second order FP,p1=0, p2=1	4151.56
Second order FP,p1=0, p2=2	4561.43
Second order FP,p1=0, p2=3	4809.67
Second order FP,p1=0.5, p2=0.5	4144.61
Second order FP,p1=0.5, p2=1	4371.3
Second order FP,p1=0.5, p2=2	4690.99
Second order FP,p1=0.5, p2=3	4866.07
Second order FP,p1=1, p2=1	4547.61
Second order FP,p1=1, p2=2	4766.54
Second order FP,p1=1, p2=3	4874.75

Second order FP,p1=2, p2=2	4836.2
Second order FP,p1=2, p2=3	4878.64
Second order FP,p1=3, p2=3	4913.18

Table S6 AIC value for OS fit

Fractional Polynomial	AIC Value
First order FP,p1=0	5169.32
First order FP,p1=1	5056.42
First order FP,p1=-2	4879.68
First order FP,p1=-1	5083.71
First order FP,p1=-0.5	5160.72
First order FP,p1=0.5	5118.22
First order FP,p1=2	4990.78
First order FP,p1=3	4983.29
Second order FP,p1=-2, p2=-2	4287.36
Second order FP,p1=-2, p2=-1	4245.83
Second order FP,p1=-2, p2=-0.5	4231.87
Second order FP,p1=-2, p2=0	4230.89
Second order FP,p1=-2, p2=0.5	4245.99
Second order FP,p1=-2, p2=1	4274.3
Second order FP,p1=-2, p2=2	4347
Second order FP,p1=-2, p2=3	4417.3
Second order FP,p1=-1, p2=-1	4250.92
Second order FP,p1=-1, p2=-0.5	4270.01
Second order FP,p1=-1, p2=0	4303.51
Second order FP,p1=-1, p2=0.5	4349.49
Second order FP,p1=-1, p2=1	4402.38
Second order FP,p1=-1, p2=2	4506.81
Second order FP,p1=-1, p2=3	4594.24
Second order FP,p1=-0.5, p2=-0.5	4309.02
Second order FP,p1=-0.5, p2=0	4361.63
Second order FP,p1=-0.5, p2=0.5	4423.45
Second order FP,p1=-0.5, p2=1	4487.82
Second order FP,p1=-0.5, p2=2	4604.32
Second order FP,p1=-0.5, p2=3	4695.63
Second order FP,p1=0, p2=0	4431.37
Second order FP,p1=0, p2=0.5	4505.95
Second order FP,p1=0, p2=1	4577.85
Second order FP,p1=0, p2=2	4697.16
Second order FP,p1=0, p2=3	4783.52
Second order FP,p1=0.5, p2=0.5	4587.95
Second order FP,p1=0.5, p2=1	4661.42
Second order FP,p1=0.5, p2=2	4772.27
Second order FP,p1=0.5, p2=3	4845.1
Second order FP,p1=1, p2=1	4730.92
Second order FP,p1=1, p2=2	4825.78
Second order FP,p1=1, p2=3	4882.29

Second order FP,p1=2, p2=2	4886.95
Second order FP,p1=2, p2=3	4919.54
Second order FP,p1=3, p2=3	4940.54

Table S7 Hazard function of PFS for each of the 10 immunotherapy regimens

	Treatment	Ln h(t)
1	nivo+ipi+chemo	$-3.9076-33.3927t^{-2}+16.2076t^{-1}$
2	nivo	$-4.3777-59.6317t^{-2}+26.2663t^{-1}$
3	nivo+ipi	$-4.6348-59.2767t^{-2}+26.3614t^{-1}$
4	nivo+chemo	$-4.1633-82.6963t^{-2}+26.0447t^{-1}$
5	ate	$-3.8169-28.6951t^{-2}+15.456t^{-1}$
6	ate+chemo	$-2.9073-21.0096t^{-2}+7.6194t^{-1}$
7	ate+beva+chemo	$-2.286-4.5066t^{-2}-0.9354t^{-1}$
8	pem+chemo	$-3.6553-58.0449t^{-2}+16.6701t^{-1}$
9	pem+ipi	$-3.3312-21.5776t^{-2}+11.2562t^{-1}$
10	pem	$-3.496-24.9415t^{-2}+13.2047t^{-1}$

Table S8 Hazard function of PFS for each of the 4 PD-L1 expression levels

	PD-L1	Ln h(t)
1	>=1%	$-3.679-28.4848t^{-2}+14.6504t^{-1}$
2	>=1% and <50%	$-3.3467-25.4273t^{-2}+12.9039t^{-1}$
3	>=50%	$-3.8723-24.7631t^{-2}+13.5697t^{-1}$
4	<1%	$-3.2645-24.0279t^{-2}+12.2456t^{-1}$
5	all	$-3.496-24.9415t^{-2}+13.2047t^{-1}$

Table S9 Hazard function of OS for each of the 10 immunotherapy regimens

	Treatment	Ln h(t)
1	nivo+ipi+chemo	$-2.48352-7.33101t^{-2}-0.24904\log(t)$
2	nivo	$-1.30505-16.70896t^{-2}-0.6201\log(t)$
3	nivo+ipi	$-0.94748-18.75276t^{-2}-0.83209\log(t)$
4	nivo+chemo	$-1.46273-24.37577t^{-2}-0.58251\log(t)$
5	ate	$-2.29041-5.1484t^{-2}-0.34366\log(t)$
6	ate+chemo	$-2.29691-8.14711t^{-2}-0.34832\log(t)$
7	ate+beva+chemo	$-3.33785-2.7853t^{-2}-0.00881\log(t)$
8	pem+chemo	$-2.11355-21.09752t^{-2}-0.42402\log(t)$
9	pem+ipi	$-2.31789-5.75427t^{-2}-0.33122\log(t)$
10	pem	$-1.52234-12.99996t^{-2}-0.56231\log(t)$

Table S10 Hazard function of OS for each of the 4 PD-L1 expression levels

	PD-L1	Ln h(t)
1	>=1%	$-1.43457-12.94573t^{-2}-0.6367\log(t)$
2	>=1% and <50%	$-1.24162-15.54684t^{-2}-0.62334\log(t)$
3	>=50%	$-1.82544-9.49396t^{-2}-0.58377\log(t)$
4	<1%	$-1.67197-12.48552t^{-2}-0.45131\log(t)$
5	all	$-1.52234-12.99996t^{-2}-0.56231\log(t)$

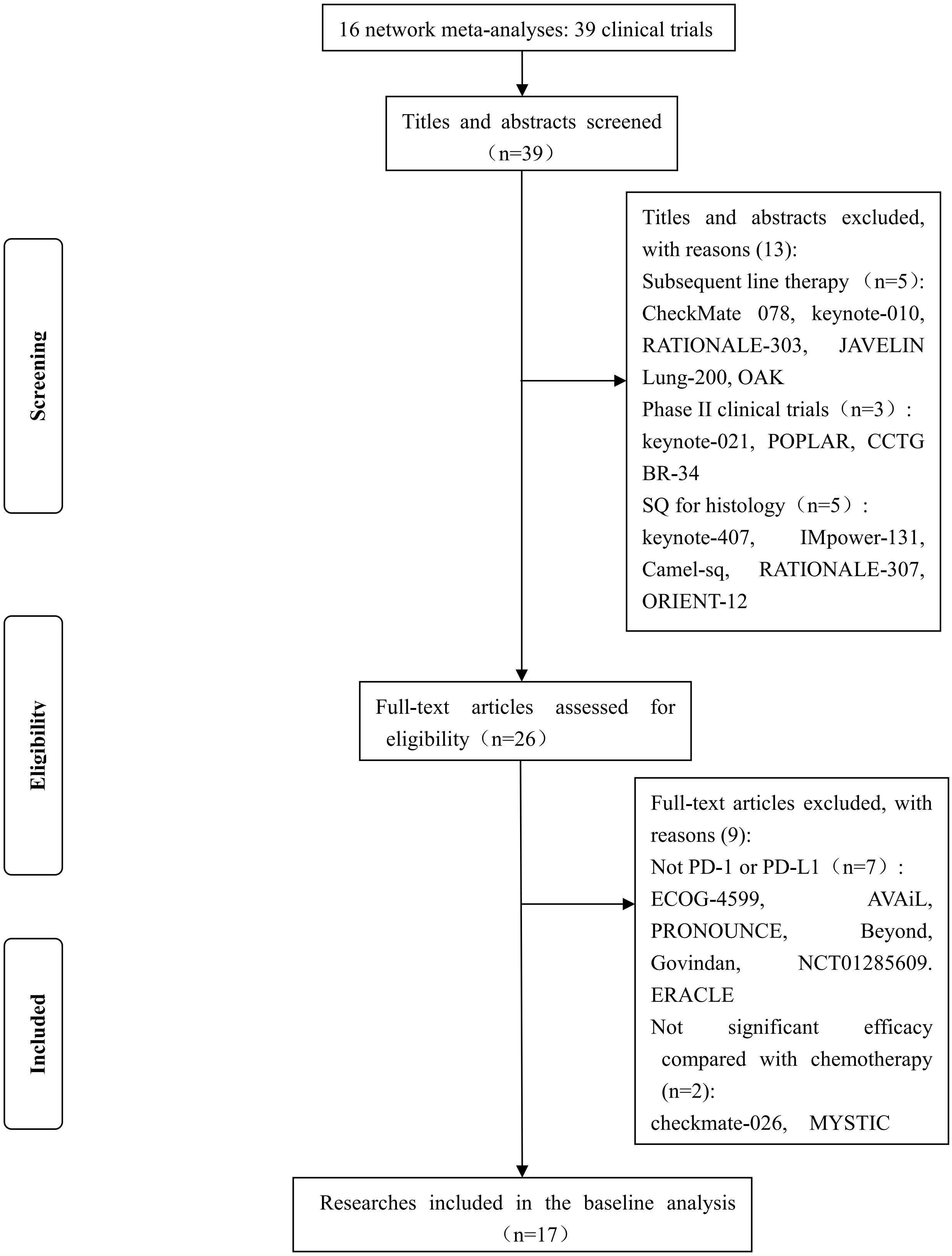

Figure S1. Flow diagram for the clinical trial selection procedure

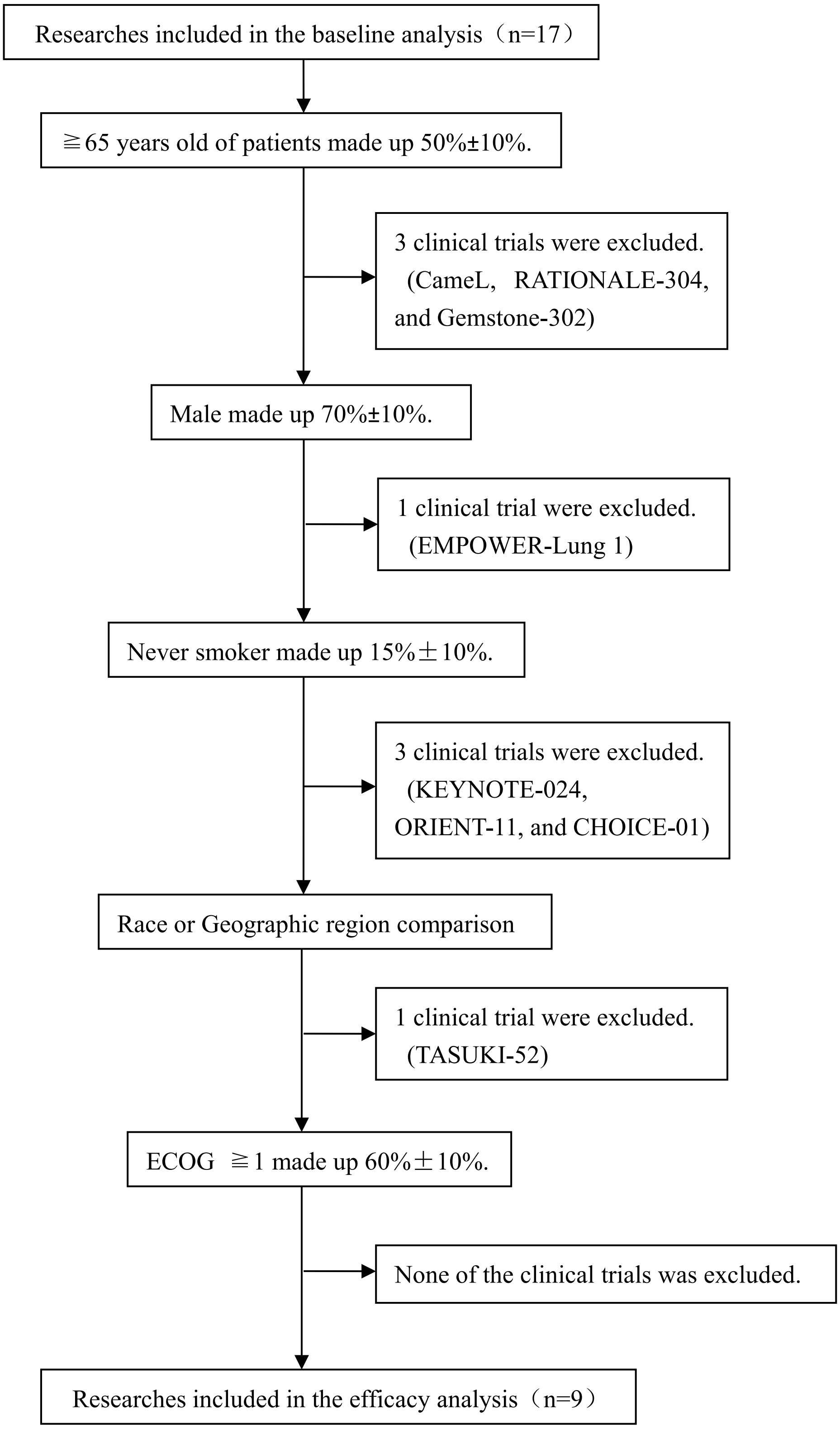

Figure S2. Flow diagram for the baseline characteristic balance

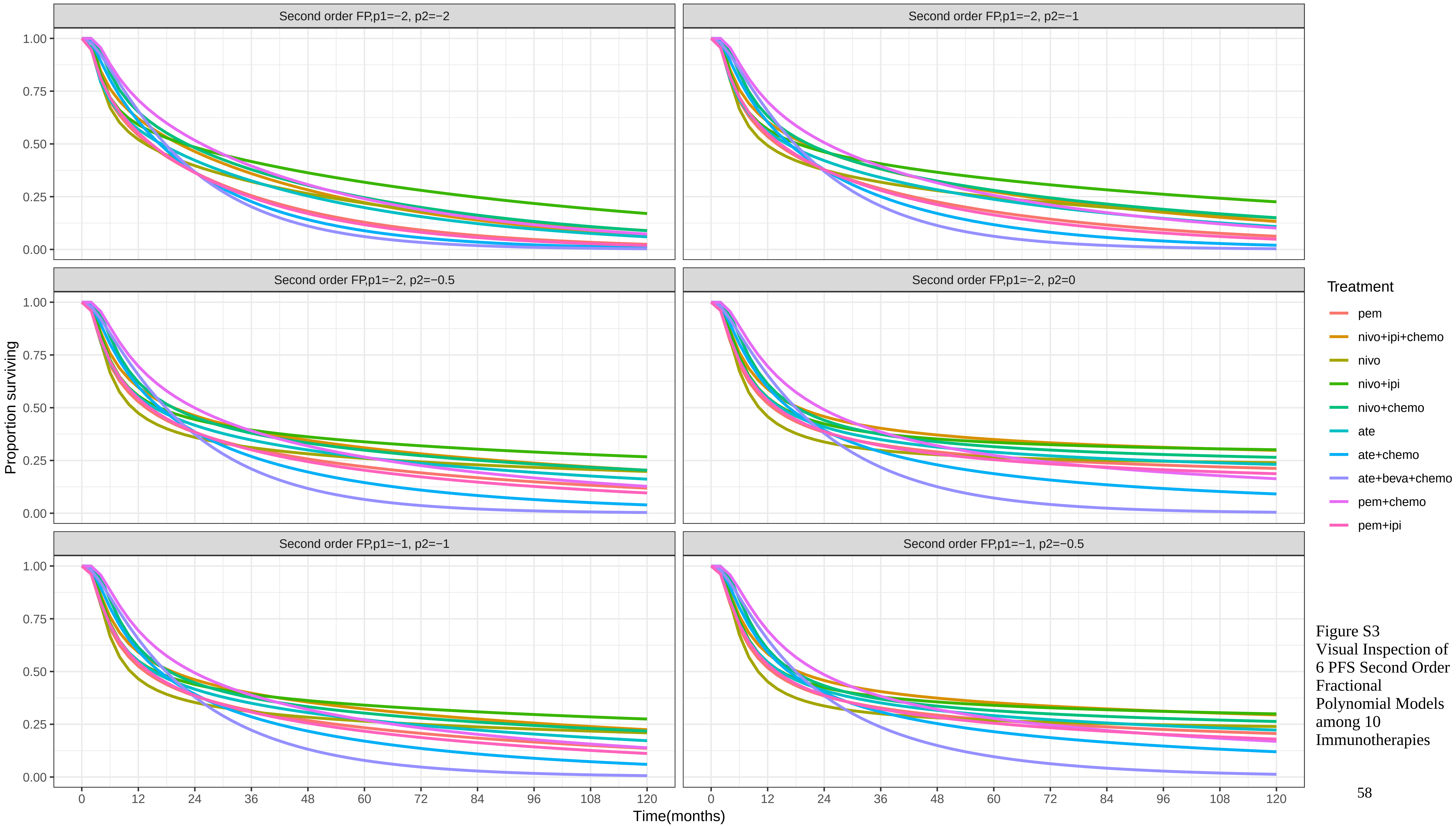

Figure S3
Visual Inspection of 6 PFS Second Order Fractional Polynomial Models among 10 Immunotherapies

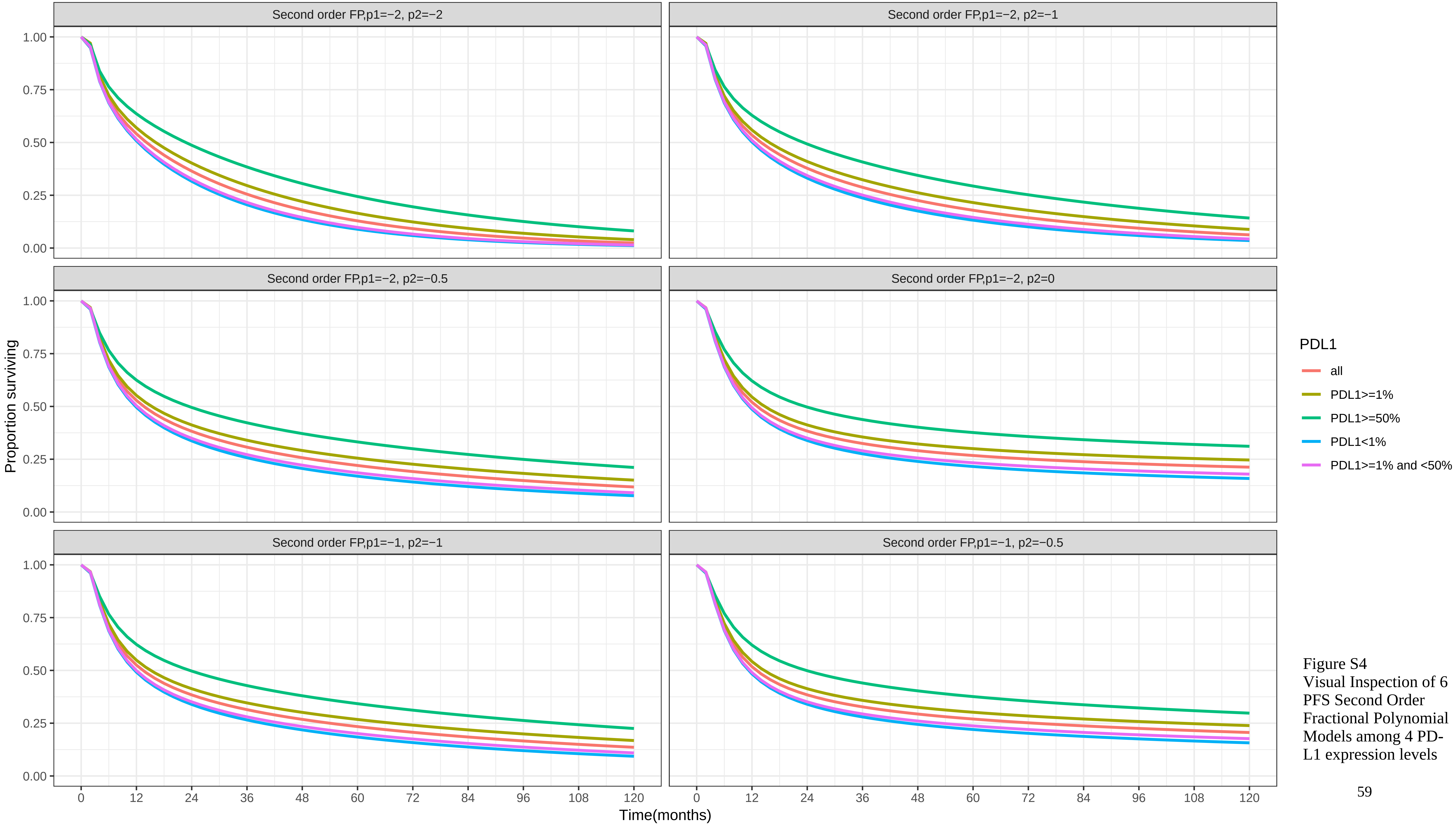

Figure S4
Visual Inspection of 6 PFS Second Order Fractional Polynomial Models among 4 PD-L1 expression levels

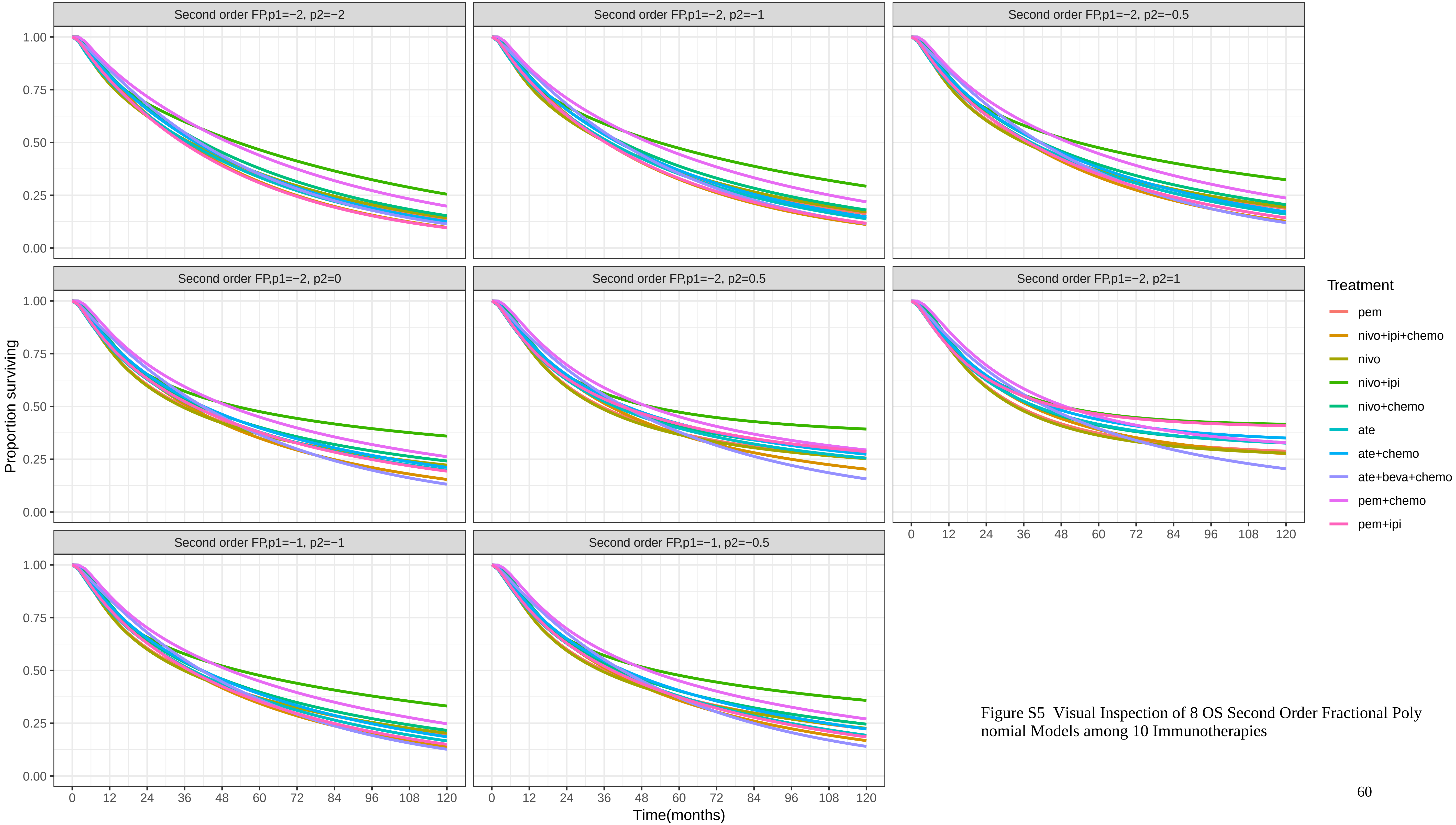

Figure S5 Visual Inspection of 8 OS Second Order Fractional Poly nomial Models among 10 Immunotherapies

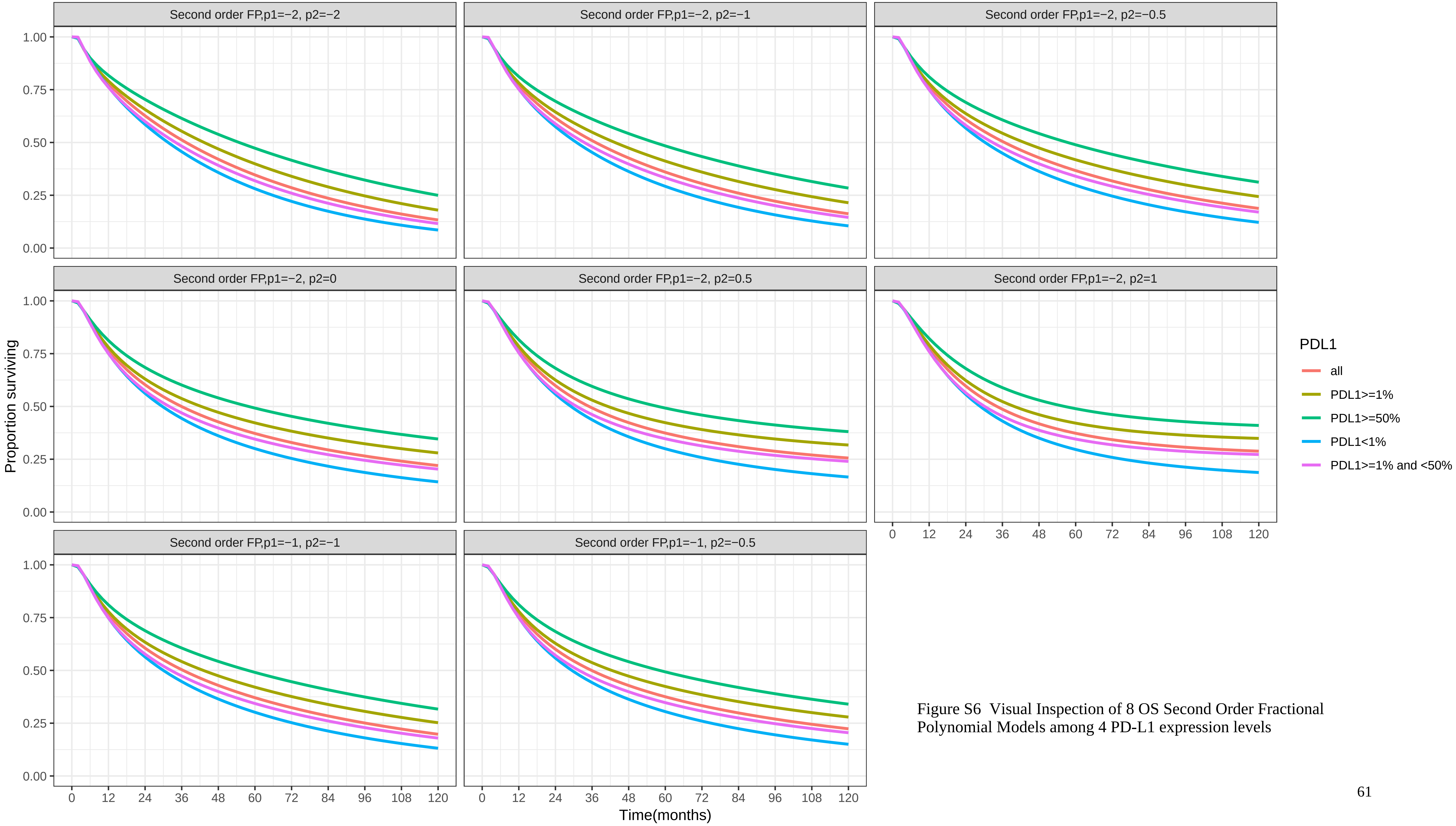

Figure S6 Visual Inspection of 8 OS Second Order Fractional Polynomial Models among 4 PD-L1 expression levels
